# Changes in prices, sales, consumer spending, and beverage consumption one year after a tax on sugar-sweetened beverages in Berkeley, California, US: A before-and-after study

**DOI:** 10.1371/journal.pmed.1002283

**Published:** 2017-04-18

**Authors:** Lynn D. Silver, Shu Wen Ng, Suzanne Ryan-Ibarra, Lindsey Smith Taillie, Marta Induni, Donna R. Miles, Jennifer M. Poti, Barry M. Popkin

**Affiliations:** 1 Public Health Institute, Oakland, California, United States of America; 2 Department of Nutrition, University of North Carolina at Chapel Hill, Chapel Hill, North Carolina, United States of America; 3 Carolina Population Center, University of North Carolina at Chapel Hill, Chapel Hill, North Carolina, United States of America; University of Cambridge, UNITED KINGDOM

## Abstract

**Background:**

Taxes on sugar-sweetened beverages (SSBs) meant to improve health and raise revenue are being adopted, yet evaluation is scarce. This study examines the association of the first penny per ounce SSB excise tax in the United States, in Berkeley, California, with beverage prices, sales, store revenue/consumer spending, and usual beverage intake.

**Methods and findings:**

Methods included comparison of pre-taxation (before 1 January 2015) and first-year post-taxation (1 March 2015–29 February 2016) measures of (1) beverage prices at 26 Berkeley stores; (2) point-of-sale scanner data on 15.5 million checkouts for beverage prices, sales, and store revenue for two supermarket chains covering three Berkeley and six control non-Berkeley large supermarkets in adjacent cities; and (3) a representative telephone survey (17.4% cooperation rate) of 957 adult Berkeley residents.

Key hypotheses were that (1) the tax would be passed through to the prices of taxed beverages among the chain stores in which Berkeley implemented the tax in 2015; (2) sales of taxed beverages would decline, and sales of untaxed beverages would rise, in Berkeley stores more than in comparison non-Berkeley stores; (3) consumer spending per transaction (checkout episode) would not increase in Berkeley stores; and (4) self-reported consumption of taxed beverages would decline.

Main outcomes and measures included changes in inflation-adjusted prices (cents/ounce), beverage sales (ounces), consumers’ spending measured as store revenue (inflation-adjusted dollars per transaction) in two large chains, and usual beverage intake (grams/day and kilocalories/day).

Tax pass-through (changes in the price after imposition of the tax) for SSBs varied in degree and timing by store type and beverage type. Pass-through was complete in large chain supermarkets (+1.07¢/oz, *p* = 0.001) and small chain supermarkets and chain gas stations (1.31¢/oz, *p* = 0.004), partial in pharmacies (+0.45¢/oz, *p* = 0.03), and negative in independent corner stores and independent gas stations (−0.64¢/oz, *p* = 0.004). Sales-unweighted mean price change from scanner data was +0.67¢/oz (*p* = 0.00) (sales-weighted, +0.65¢/oz, *p* = 0.003), with +1.09¢/oz (*p <* 0.001) for sodas and energy drinks, but a lower change in other categories. Post-tax year 1 scanner data SSB sales (ounces/transaction) in Berkeley stores declined 9.6% (*p <* 0.001) compared to estimates if the tax were not in place, but rose 6.9% (*p <* 0.001) for non-Berkeley stores. Sales of untaxed beverages in Berkeley stores rose by 3.5% versus 0.5% (both *p <* 0.001) for non-Berkeley stores. Overall beverage sales also rose across stores. In Berkeley, sales of water rose by 15.6% (*p <* 0.001) (exceeding the decline in SSB sales in ounces); untaxed fruit, vegetable, and tea drinks, by 4.37% (*p <* 0.001); and plain milk, by 0.63% (*p* = 0.01). Scanner data mean store revenue/consumer spending (dollars per transaction) fell 18¢ less in Berkeley (−$0.36, *p <* 0.001) than in comparison stores (−$0.54, *p <* 0.001). Baseline and post-tax Berkeley SSB sales and usual dietary intake were markedly low compared to national levels (at baseline, National Health and Nutrition Examination Survey SSB intake nationally was 131 kcal/d and in Berkeley was 45 kcal/d). Reductions in self-reported mean daily SSB intake in grams (−19.8%, *p* = 0.49) and in mean per capita SSB caloric intake (−13.3%, *p* = 0.56) from baseline to post-tax were not statistically significant.

Limitations of the study include inability to establish causal links due to observational design, and the absence of health outcomes. Analysis of consumption was limited by the small effect size in relation to high standard error and Berkeley’s low baseline consumption.

**Conclusions:**

One year following implementation of the nation’s first large SSB tax, prices of SSBs increased in many, but not all, settings, SSB sales declined, and sales of untaxed beverages (especially water) and overall study beverages rose in Berkeley; overall consumer spending per transaction in the stores studied did not rise. Price increases for SSBs in two distinct data sources, their timing, and the patterns of change in taxed and untaxed beverage sales suggest that the observed changes may be attributable to the tax. Post-tax self-reported SSB intake did not change significantly compared to baseline. Significant declines in SSB sales, even in this relatively affluent community, accompanied by revenue used for prevention suggest promise for this policy. Evaluation of taxation in jurisdictions with more typical SSB consumption, with controls, is needed to assess broader dietary and potential health impacts.

## Abstract 2

The effects of a sugar-sweetened tax on beverages in Berkeley, USA are revealed by Barry Popkin and colleagues. In this before and after analysis set in an affluent area, sales of sweetened beverages fall.

## Author summary

**Why was this study done?:**

- Berkeley passed the first large (one cent per ounce) tax on sugar-sweetened beverages (SSBs) in the United States in November 2014, affording a unique opportunity for evaluation.
- It was unknown to what extent people would change beverage purchasing in response to a tax, especially in a relatively prosperous community.
- Few high-quality evaluations from other countries existed; studies of Mexico’s tax (implemented starting January 2014) found substantial but not complete “pass-through” of the tax to consumers for taxed beverages, a 6% reduction in sales in the first year, and a 9% decrease in sales to lower-income households.

**What did the researchers do and find?:**

- Three before-and-after studies were carried out: one of store scanner records from 15.5 million checkouts in two chains of large groceries in Berkeley and comparison cities; one of 26 stores of various types in Berkeley; and one random digit dialing telephone survey of consumption by Berkeley residents.
- In the 15.5 million supermarket checkouts studied, 67% of the amount of the tax was passed on to consumers across all SSBs, and the tax was fully passed through for sodas and energy drinks; in the 26-store survey, the tax was more than fully passed on in Berkeley large and small chain groceries and gas stations, especially for carbonated beverages; partially passed on in pharmacies; and not passed on in small independent gas stations and corner stores.
- Sales in ounces of taxed SSBs fell by 9.6% in relation to predicted sales in the absence of the tax, while sales of untaxed beverages rose 3.5% and total beverage sales rose in Berkeley. Consumer spending per transaction (average grocery bill) did not increase, nor did store revenue fall more in Berkeley, while SSB sales rose 6.9% in comparison cities.
- Berkeley residents were low consumers of SSBs at baseline (consuming only 34% of the national average of SSBs). Dietary intake surveys found shifts of −19.8% (*p* = 0.49) in mean daily SSB intake (grams) and −13.3% (*p* = 0.56) in mean calories from SSBs that were not statistically significant, while caloric intake of untaxed beverages (milk and other diary-based beverages) increased.

**What do these findings mean?:**

- Berkeley’s innovative tax on SSBs was mostly, though not uniformly, passed through to consumers, and sales of SSBs declined significantly, consistent with published price elasticity estimates.
- There was no evidence in studied chains of higher grocery bills for consumers, loss of gross revenue per transaction for stores, or decreases in overall beverage sales for stores. While telephone respondents did not report changes in shopping location, scanner data were consistent with some increased purchasing of SSBs in neighboring cities.
- The findings of this study, while limited by its observational design, suggest that SSB taxes may be effective in shifting consumers to purchase healthier beverages without causing undue economic hardship and while raising revenue for social objectives.
- Population-based findings on SSB consumption were not definitive, and consumption should be further evaluated in more typical communities and with larger samples.

## Introduction

Sugar-sweetened beverage (SSB) consumption is linked to increased body weight, diabetes, cardiovascular risk factors, and dental caries, amongst other conditions [1,2]. Significant SSB taxes have been proposed and increasingly adopted as part of a comprehensive approach to obesity and diabetes prevention [3–5] with extensive potential health and social benefits [2,5–7]. Over 20 countries have passed strengthened SSB taxes of varying sizes, with a growing emphasis on larger excise taxes [6,8–10].

Berkeley, California, is the first US jurisdiction to successfully place a substantial excise tax on SSB distributors, with the dual goals of reducing consumption and raising revenue for efforts to prevent obesity and diabetes. The tax, approved by voters in November 2014, is one penny per fluid ounce (1¢/oz) on beverages with added caloric sweeteners. In theory, the tax might add 68¢ to the price of a 2-l (68-oz) bottle of soda, typically priced a little over $2 before the tax, or 12¢ to a 12-oz can, sold for around $1. In late January 2015, the city delayed the original 1 January 2015 implementation until 1 March 2015 among the 38 largest beverage distributors [11]. Tax collection from small retailers obtaining their own supplies (“self-distributors”) only began 1 January 2016. In 2016, other US jurisdictions, including three large cities—Philadelphia (Pennsylvania), San Francisco (California), and Oakland (California)—and Cook County (Illinois), which encompasses Chicago’s metro area, as well as two smaller cities—Boulder (Colorado) and Albany (California)—followed suit, with similar measures at tax levels from 1¢/oz to 2¢/oz.

The Berkeley tax therefore offered a unique opportunity to evaluate this policy. This study sought to examine (1) whether and how the tax was passed through to beverage prices, (2) whether the volume of beverages sold changed, (3) whether store revenues/consumer spending per transaction within these stores changed, and (4) whether beverage consumption changed. This study evaluates changes in the first year of implementation (March 2015–February 2016).

## Methods

**Fig 1:**
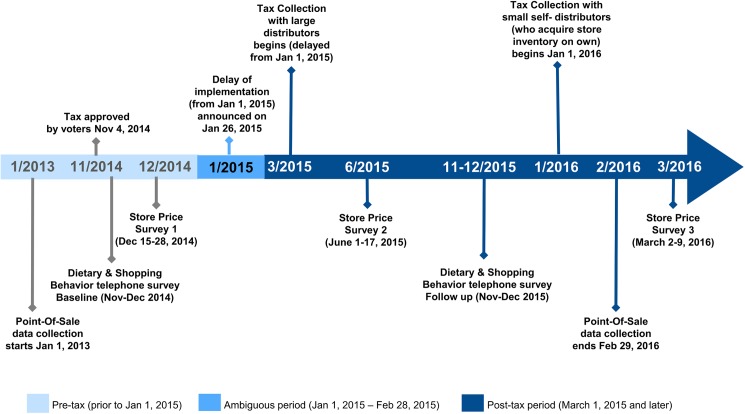
Berkeley sugar-sweetened beverage tax implementation and evaluation timeline.

Three data collection approaches were employed to measure beverage prices, volume sold, store revenue (or, conversely, consumer spending), and beverage intake. Fig 1 illustrates the tax implementation and study data collection timeline. Key elements of analyses were determined prospectively; however, some adjustments were required, particularly as we received and analyzed store scanner data.

This study was approved by institutional review boards of the Public Health Institute and the University of North Carolina at Chapel Hill.

**Setting.** The city of Berkeley, located in California’s Bay Area, had an estimated 121,000 inhabitants in 2015 and covers only 10.5 square miles. Residents are 55% non-Hispanic white, 19% Asian, 11% Hispanic or Latino, 10% African-American, and 21% foreign-born. Berkeley is home to a large public university and a very highly educated population, with 71% of those over age 25 y holding a bachelor’s degree or higher. Nevertheless, it has a high percentage of residents in poverty (20.4% versus 15.3% for California and 13.5% nationwide), though the median income of $66,237 is about 10% above the median for the state as a whole and 23% above the US median [12].

**Store price surveys.** Store price surveys were conducted in December 2014 (pre-tax), June 2015 (4 months post-tax), and March 2016 (13 months post-tax, and 2 months into self-distributor tax collection) among a targeted sample of large supermarkets, small chain supermarkets, chain and independent gas stations, pharmacies (drugstores), and independent corner stores located in Berkeley, California (*n* = 26). Six top stores were identified from the telephone survey (described below), and the remainder were selected randomly within their type. Store price surveys collected 744 prices in December 2014, 798 prices in June 2015, and 633 prices in March 2016 for a standard panel of 70 beverages, which included 45 taxed and untaxed branded beverages in a variety of sizes. It was possible to collect 313 prices for 55 of the 70 products in the standard panel in all three rounds in the same stores. S1 Text provides details on the store price survey design.

**Point-of-sale data.** Point-of-sale electronic scanner data were requested using personal outreach to all large supermarkets in Berkeley, as well as to pharmacies, small supermarkets, ethnic markets, convenience stores, and gas stations with scanner systems, and with extensive follow-up as needed to owners or corporate headquarters. Ultimately, two chains of large supermarkets with three of the city’s nine large groceries provided electronic data covering 1 January 2013 through 29 February 2016 (26 months pre-tax; 12 months post-tax). They also provided data on six Bay Area control stores. Data covered 118.8 million barcode scans from 15.5 million transactions (checkout episodes), with 16.2 million barcode scans involving beverages (16,769 unique barcodes), of which 10.8 million barcode scans (5,631 unique barcodes) are included here. S2 Text describes the point-of-sale study design and the stores and beverage products included in our analyses. The tax status of each beverage was classified using the Berkeley law [13], nutrition data from product websites, and ingredient data from Mintel [14].

**Dietary and shopping behavior surveys.** These telephone surveys were conducted November–December 2014 (pre-tax/baseline) and November–December 2015 (post-tax/follow-up). The sample was identified using dual frame (landline/cellular) random digit dialing that oversampled lower income census blocks (>50% of households with annual gross household income <$100,000) in Berkeley. Only Berkeley residents were interviewed. Oral informed consent was obtained from all participants. Trained interviewers used standardized questionnaires and computer-assisted telephone interviews to collect information on beverage shopping locations and behaviors, demographics, and 24-h recall of beverage intake [15]. To adjust for typical daily intake, a second 24-h beverage recall interview was collected 3–7 d later from consenting respondents.

Sampling weights were calculated using iterative proportional fitting (raking) [16] to adjust the data to demographic proportions for Berkeley, California, obtained from the United States Census Bureau for 2010 [12]. Details on the sample design, other methods, and response rates are found in S5 Text. Caloric intake from beverages consumed was calculated using nutrition data from product websites, nutrition facts panel data from Mintel [14], and US Department of Agriculture databases [17,18].

### Analytical approaches

#### Changes in prices

Prices were calculated based on prices paid, excluding sales tax and California Redemption Value bottle fee. Inflation-adjusted prices were derived by applying the US Bureau of Labor Statistics Consumer Price Index (CPI) for the monthly average price of non-alcoholic beverages [19] to price measures, using January 2013 as the base. To measure changes in price after imposition of the tax, known as “pass-through,” using prices from the store price surveys, we compared the mean prices in cents/ounce of beverage products collected across the 26 stores in Berkeley at three time points (December 2014, June 2015, and March 2016) using paired *t*-tests. Data were analyzed using only beverages that could be matched for product and size across all three rounds, reflecting same product prices, rather than total consumer experience. For details see S1 Text.

Point-of-sale data included repeated measures of beverages sold (at barcode level) at both Berkeley and non-Berkeley stores, during both pre-tax and post-tax periods (see S2 Text for details). We used a fixed effects approach using the price (cents/ounce) of taxed beverages per barcode-month-store as the outcome, controlling for month-year (relative to January 2013) and potential underreporting due to data that were missing completely at random because of technical (data storage) issues for some stores on random days that contributed to the monthly value. For model specifications, see S3 Text. From the models, adjusted beverage prices (cents/ounce) in Berkeley versus non-Berkeley stores overall and by beverage category were derived. Since the tax implementation timeline was altered, the January–February 2015 period was ambiguous with regards to tax implementation and price change, so we compared prices from March–December in 2016 to the same 10-month period in earlier years. All analyses were conducted in Stata 13 [20].

#### Changes in sales and store revenue (consumer spending)

Store-day data on the volume of taxed and untaxed beverages (ounces per transaction) and average daily store revenues (CPI-adjusted dollars per transaction) from all sales were the key outcomes and were modeled separately. We examined whether there were differences in these outcomes in non-Berkeley stores by distance from Berkeley. Comparison stores were classified into zones: zone 1, adjacent to Berkeley (two stores in two cities); zone 2, San Francisco (one store); and zone 3, ≥20 miles (three stores in three cities) (see map in S4 Text). Since the beverage volume distributions (and their residuals) were skewed, outcomes were log-transformed to normalize distributions. For the volume outcomes, ordinary least squares models were used, with controls for store ID, day of week, holiday and holiday eve, month, year, number of transactions (linear and quadratic), a post-tax indicator, and interactions of store ID with the post-tax indicator, month, and year, correcting the standard errors by clustering the analyses at the city level. A similar model was used for revenue per transaction (a measure of the gross revenue for the stores as well as customer’s spending in these stores), excluding number of transactions as a control.

To test whether the post-tax trend in sales differed significantly from the pre-tax trend, we predicted taxed and untaxed beverage sale volume and store revenue per transaction if the post-tax indicator = 0 during March 2015–February 2016 (i.e., a “counterfactual” for if the tax had not been implemented [9]) and compared these predicted values to the adjusted volumes observed during the post-tax period. For detailed specifications, see S4 Text.

#### Changes in usual intake of beverages

Using a repeated cross-sectional approach, the National Cancer Institute method was used to estimate the usual intake distribution (kilocalories/day and grams/day) of taxed and untaxed beverages in each year, controlling for age, gender, race/ethnicity, education, income, weekend (including Friday), and recall sequence [21,22]. To account for the large proportion of nonconsumers for taxed beverages, a two-part probability-amount nonlinear mixed model was fitted [23,24], while a one-part nonlinear mixed model was fitted for untaxed beverages. Standard errors were estimated via bootstrapping, with 200 replications. The primary outcomes of interest were change in calories and grams consumed from taxed beverages, using a two-sided test with statistical significance set at *p <* 0.05. For modeling approach, see S6 Text.

## Results

### Store price survey prices

**Fig 2:**
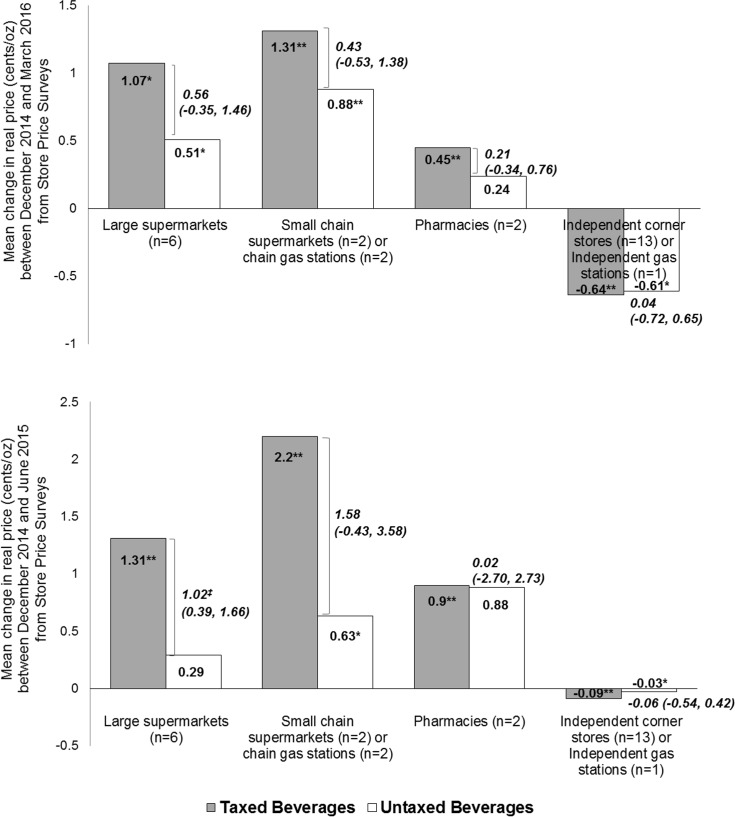
Store price survey mean (95% CI) beverage price changes (cents per ounce) in Berkeley stores. Top: price change between December 2014 (round 1) and March 2016 (round 3). Bottom: price change between December 2014 (round 1) and June 2015 (round 2). Sample limited to 55 product types with 313 prices across stores that were collected in all three rounds of the store price survey; of these, 56% were prices for taxed beverages and 44% for untaxed beverages. Prices account for inflation. Values in bold italics show the price difference between taxed and untaxed beverages. *Statistically significant difference between prices in later round (March 2016 or June 2015) compared to December 2014 at *p <* 0.05 using paired *t*-tests. **Statistically significant difference between prices in later round (March 2016 or June 2015) compared to December 2014 at *p <* 0.01 using paired *t*-tests. ^‡^Statistically significant difference of price of taxed beverages compared to untaxed beverages at *p <* 0.05 (unpaired *t*-tests since taxed and untaxed beverage items are different). Source: store price survey data collected by Public Health Institute.

Prices of taxed beverages collected in all three time points across large supermarkets showed increases from December 2014 (pre-tax) to June 2015 (1.31¢/oz), which continued for December 2014 to March 2016 (1.07¢/oz). Taxed beverages in small chain supermarkets and gas stations also had price increases from December 2014 to June 2015 (2.20¢/oz) that continued through March 2016 (1.31¢/oz). Price increases were comparatively lower in pharmacies for both intervals (0.90¢/oz and 0.45¢/oz) and were not seen in independent corner stores and gas stations (−0.09¢/oz and −0.64¢/oz) (S3 Table). The difference between prices of taxed and untaxed beverages (cents/ounce) increased in all store types between December 2014 and March 2016, except for beverages sold in independent corner stores and gas stations (Fig 2).

### Point-of-sale prices from two supermarket chains

**Fig 3:**
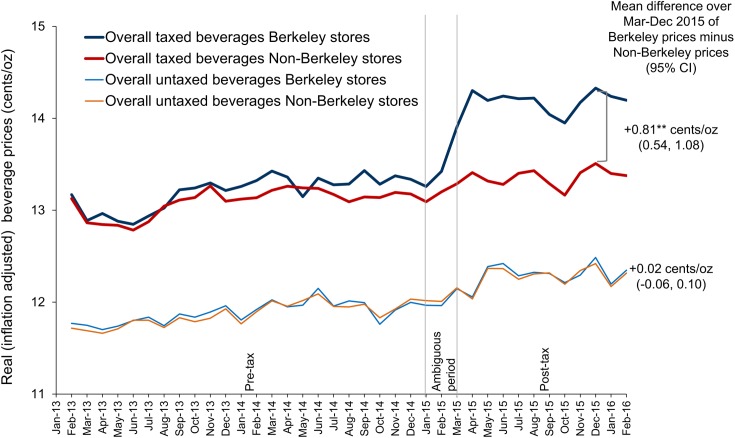
Point-of-sale model adjusted beverage prices (cents per ounce) in Berkeley versus non-Berkeley stores (sales unweighted). Fixed effects models account for the month-year (indicator variables), store located or not located in Berkeley, interaction of Berkeley store and month-year, and an indicator variable of underreported sales data from each store in particular month. Prices account for inflation. Vertical lines demarcate the pre-tax period (January 2013–December 2014), the ambiguous period (January–February 2015), and the post-tax period (March 2015–February 2016). Full sales-unweighted results can be found in S8 Table. Full sales-weighted results can be found in S9 Table. **Statistically significant difference between the Berkeley and non-Berkeley prices for March–December 2015 at *p <* 0.01. Source: point-of-sale data from two chains of large supermarkets in the Bay Area obtained by the Public Health Institute.

Fig 3 shows the model adjusted sales-unweighted beverage prices in Berkeley and non-Berkeley stores, illustrating the price differential for taxed versus untaxed beverages and change in prices of taxed beverages over time. Among taxed beverages, there were visible price increases in Berkeley stores after January 2015, but it was not until around April 2015 that prices stabilized. Specifically, among the Berkeley stores, taxed beverages had price change of +0.83¢/oz (*p <* 0.001), while this was only +0.16¢/oz (*p <* 0.001) in non-Berkeley stores, for a net difference of +0.67¢/oz (*p <* 0.001). Meanwhile, there were no statistically significant differences in the prices of untaxed beverages between Berkeley and non-Berkeley stores in the post-tax period. Sales-unweighted pass-through was complete among sodas and energy drinks (+1.09¢/oz), but incomplete for the other taxed beverage groups (S8 Table). Sales-weighted price changes for taxed beverages was similar, at +0.69¢/oz (S9 Table).

### Point-of-sale volume sold in two supermarket chains

**Fig 4:**
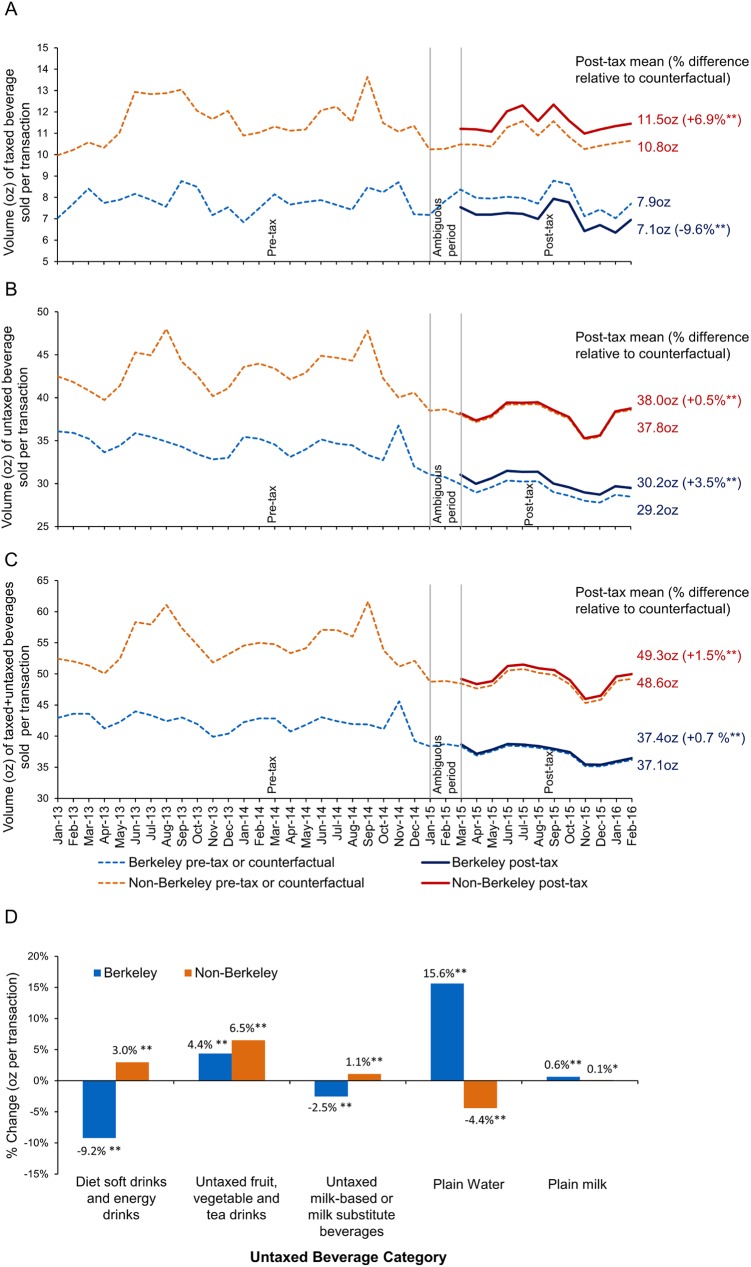
Point-of-sale adjusted mean daily volume of beverages sold (ounces per transaction) in Berkeley versus non-Berkeley stores. (A) Point-of-sale taxed beverage volume sold (ounces per transaction).(B) Point-of-sale untaxed beverage volume sold (ounces per transaction).(C) Point-of-sale taxed and untaxed beverage volume sold (ounces per transaction).(D) Percent change in post-tax untaxed beverage sales (ounces per transaction) in relation to counterfactual in Berkeley and non-Berkeley stores. Models account for store ID, month, year, day of week, holiday and holiday eve, number of transactions (linear and quadratic), a post-tax indicator, and interactions of store ID with the post-tax indicator, month, and year variables, correcting the standard errors by clustering the analyses at the city level. Back-transformation uses Duan smearing. Model *n* = 10,152. Vertical lines demarcate the pre-tax period (January 2013–December 2014), the ambiguous period (January–February 2015), and the post-tax period (March 2015–February 2016). To derive the counterfactuals, we predicted the volume of taxed and untaxed beverages sold if the post-tax indicator = 0 for March 2015–February 2016. Full results can be found in S10 and S11 Tables. *Statistically significant difference between the counterfactual and observed volumes sold during the entire post-tax period at *p <* 0.05. **Statistically significant difference between the counterfactual and observed volumes sold during the entire post-tax period at *p <* 0.01. Source: point-of-sale data from two chains of large supermarkets in the Bay Area obtained by the Public Health Institute.

The volume of untaxed beverages sold was consistently higher than for taxed beverages in all locations January 2013–February 2016 (Fig 4), and both types of sales were consistently and markedly lower in Berkeley than in comparison stores overall, and most notably in neighboring zone 1 stores, suggesting lower baseline purchasing of SSBs and of beverages in general.

Focusing on the post-tax period, our model adjusted estimates show that compared to their counterfactuals, volume (ounces/transaction) of taxed beverages sold fell significantly by 9.6% in Berkeley stores, but rose by 6.9% in non-Berkeley stores (Fig 4A), sales of untaxed beverages rose by 3.5% in Berkeley stores and 0.5% in non-Berkeley stores (Fig 4B), and sales of all study beverages increased by 0.7% and 1.5% in Berkeley and non-Berkeley stores, respectively (Fig 4C). In Berkeley, sales of untaxed water rose by 15.6%; untaxed fruit, vegetable, and tea drinks, by 4.37%; and plain milk, by 0.63%. Sales of diet soft drinks and energy drinks declined by 9.2% compared to their counterfactuals (Fig 4D).

S10 Table provides the absolute (ounces/transaction) and relative (percent) differences between the counterfactual and post-tax monthly beverage sales in Berkeley versus non-Berkeley stores overall and in the three non-Berkeley zones. Neighboring non-Berkeley stores (zone 1) had the highest increase in sales of taxed and untaxed beverages, whereas sales of taxed beverages declined in more distant zone 3. S11 Table shows the results by untaxed beverage category for Berkeley versus non-Berkeley stores.

### Point-of-sale store revenue (consumer spending) per transaction in two supermarket chains

**Fig 5:**
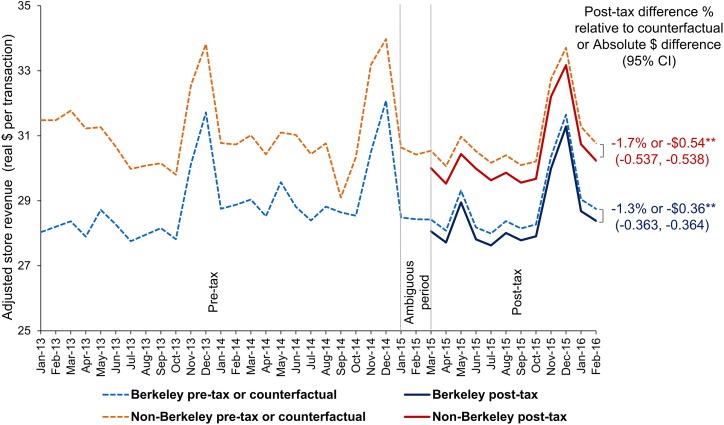
Point-of-sale adjusted mean store revenue/consumer spending (dollars per transaction) in Berkeley versus non-Berkeley stores. Models account for store ID, month, year, day of week, holiday and holiday eve, a post-tax indicator, and interactions of store ID with the post-tax indicator, month, and year variables, correcting the standard errors by clustering the analyses at the city level. Revenues account for inflation. Vertical lines demarcate the pre-tax period (January 2013–December 2014), the ambiguous period (January–February 2015), and the post-tax period (March 2015–February 2016). To derive the counterfactuals, we predicted the volume of taxed and untaxed beverages sold if the post-tax indicator = 0 in March 2015–February 2016. **Statistically significant difference between the Berkeley and non-Berkeley store revenues during the post-tax period at *p <* 0.01. Source: point-of-sale data from two chains of large supermarkets in the Bay Area obtained by the Public Health Institute.

Over the first year of the SSB tax, across both comparison and Berkeley stores, there was a small reduction in revenue in CPI-adjusted dollars per transaction from all sources (not just beverages). Mean store revenue per transaction fell by 18¢ less in Berkeley stores (−$0.36, *p <* 0.001) compared to non-Berkeley stores (−$0.54, *p <* 0.001) (see Fig 5).

### Usual intake of beverages from self-reports

At baseline (November–December 2014), 649 of 3,721 eligible and contactable Berkeley residents age ≥18 y participated (17.4% cooperation), of whom 253 completed a second 24-h beverage recall. At follow-up (November–December 2015), 654 Berkeley residents participated, and 462 completed a second 24-h beverage recall; 346 (53.3%) of the 2015 respondents had completed the baseline survey. After exclusion due to missing values on self-reported race/ethnicity, age, education, income, and monthly intake of SSBs, the final analytic sample included 623 at baseline and 613 at follow-up.

S12 Table provides details on beverage subcategories by tax status and percent consumers within subcategory before and after the tax. The Berkeley sample had lower per capita and per consumer mean caloric intake from both taxed and untaxed beverages relative to the general US population (S13 Table). At baseline, 29% of the Berkeley sample consumed SSBs, substantially below the 58% of consumers in the US population estimated in the National Health and Nutrition Examination Survey. Daily usual taxed beverage intake was 121 g/d pre-tax and 97 g/d post-tax (−13.3%, *p* = 0.49), while mean caloric intake of taxed beverages went from 45 kcal/d to 39 kcal/d (−19.8%, *p* = 0.56) (Table 1); neither difference is statistically significant. From the pre- to post-tax period, mean volume of untaxed beverage intake went from 1,839 g/d to 1,897 g/d (+3.2%, *p* = 0.21). Reported mean caloric intake of untaxed beverages rose from 116 kcal/d to 148 kcal/d (+27.6%, *p* = 0.02). The increase in untaxed calories appeared to be mainly from increased milk intake and also “other” beverages (which included dairy-based beverages such as yogurt smoothies and milkshakes). Neither juice nor diet soda intake increased. There was no significant change in reported beverage shopping location: “in Berkeley” was 90% at baseline versus 94% at follow-up (*p* = 0.17).

**Table 1:** Usual intake (kilocalories/capita/day and grams/capita/day) of beverages among adult residents of Berkeley, California, pre- and post-tax.

Usual intake	Pre-tax (Nov.–Dec. 2014), *n* = 623		Post-tax (Nov.–Dec. 2015), *n* = 613		Pre-tax–post-tax difference
	Mean	95% CI	Mean	95% CI	
**Caloric intake (kilocalories/per capita/day)**					
Taxed beverages	45.1	29.4, 60.7	38.7	23.0, 54.4	−6.4, *p* = 0.56
Untaxed beverages	115.7	87.6, 142.5	147.6	116.3, 178.9	31.9*, *p* = 0.04
**Volume of intake (grams/capita/day)**					
Taxed beverages	121.0	78.7, 163.3	97.0	56.6, 137.4	−24.0, *p* = 0.24
Untaxed beverages	1,839.4	1,692.7, 1,986.1	1,896.5	1,742.3, 2,050.8	57.1, *p* = 0.22

Models account for age, gender, race/ethnicity, income level, and educational attainment. *n* is the sample size at each round of the survey after excluding participants with missing values on self-reported race/ethnicity, age, education, income, or monthly intake of sugar-sweetened beverages.

*Statistically significant difference in mean per capita intake between pre-tax and post-tax values, *p <* 0.05.

Source: dietary and shopping behavior surveys collected by the Public Health Institute.

## Discussion

A year following SSB tax implementation in Berkeley, California, there was heterogeneous pass-through of Berkeley’s SSB excise tax across store and beverage types. SSB sales in Berkeley fell significantly in two chains of large supermarkets, while sales of untaxed beverages, especially water, and of all beverages increased. From the available data, there was no evidence of higher consumer spending, nor was there a greater reduction in store revenue per transaction in relation to comparison sites. Changes in self-reported SSB intake were not statistically significant.

For taxes to directly affect consumption, beverage distributors (upon whom the tax is levied) have to pass the tax on to retailers, and retailers likewise need to pass the tax on to consumers. Tax pass-through in the store price survey was predominant in larger and chain stores but varied in degree and implementation speed by store type, possibly reflecting delayed implementation for “self-distributing” stores. Focusing on large supermarkets, scanner data from two chains showed that the tax was partially passed through for SSBs and that pass-through varied across SSB types, being highest for carbonated beverages. This may have been due to confusion on what products were taxed, and how distributors and retailers responded to the tax based on market shares of their beverages. These findings resemble findings in Mexico [10] and France [25], where pass-through was complete on carbonated SSBs and lower on noncarbonated products. Falbe et al. [26] examined pass-through in Berkeley and comparison cities in the first 3 months post-tax, with similar findings. Cawley and Frisvold [27] examined pass-though also after only 3 months of the tax—9 months prior to full implementation of the tax in small retailers—and for a smaller panel of products (five products in several sizes each), and found lower pass-through then Falbe et al.; however, they did not have an adequate sample size of small non-chain stores [27]. The present study examined a larger group of beverage products over a full year, including the second stage of tax implementation in the third store price survey. Pass-through may still evolve, as some price changes emerged later in the year. Consumers also saw greater price differentials between taxed and untaxed beverages across all store types. Jurisdictions may wish to include recommendations to retailers in future policies to pass through the tax to SSBs. Cook County, Illinois, included a requirement to do so in their measure [28].

Despite incomplete pass-through of the SSB tax in the two chains of large supermarkets, the volume of SSBs sold fell by 9.6% in Berkeley stores. The volume of beverages sold per transaction as a whole rose in Berkeley, and shopping location did not change. This study was also unique in permitting examination of overall consumer spending at the stores studied, which did not increase, a concern widely cited by opponents of SSB taxes [29]. This study found that consumer spending, measured as store revenue per transaction, declined slightly, falling less in Berkeley stores than in comparison stores, despite increasing overall beverage sales in Berkeley. This appears to belie, at least in the chains studied, beverage industry arguments that such policies will raise grocery bills in general or that they will hurt local business. The volume of SSBs bought in stores nearest to Berkeley rose, consistent with either potential shifts to buying SSBs outside Berkeley (not reported in the telephone survey) or increasing consumption by residents of the non-Berkeley cities, as found by Falbe et al. [30]. When taxes are implemented in very small geographical areas such as Berkeley, shifts in shopping location may be a greater risk. Recent approval of similar policies in three neighboring cities may reduce or displace any shifting. Since Berkeley has on average higher education and median income and lower baseline SSB consumption compared to the US in general, it was unclear whether the tax would be high enough to change demand. In Berkeley, the post-tax sales of SSBs declined to a greater degree than in Mexico, where the decline was 6% over the first year, and this decline is consistent with earlier estimates that a 10% increase in soft drink prices would reduce consumption by 8%–12% [31,32]. The decline may be due to concomitant high rates of residents in poverty.

Using a 3- to 10-min street intercept survey of low-income residents in Berkeley and control cities, Falbe et al. found a significant 21% decline in the frequency of SSB intake in Berkeley [30]. Our telephone study used calories and grams of reported intake rather than frequency; our finding on change in mean daily SSB intake across the general population lacked statistical significance, although it was of similar magnitude to that found by Falbe et al., with a 19.8% reduction in grams. Falbe et al. examined only water consumption for untaxed beverages [30], while the present study asked about most untaxed beverages. The higher calories from untaxed beverages in our self-reported post-tax survey came predominantly from two sources: milks and “other” untaxed beverages, which included higher-fat beverages such as yogurt smoothies, milkshakes, atole, horchata, and eggnog. These findings contrast with the substitution pattern seen in our Berkeley point-of-sale data, which showed an increase in water sales and smaller but still significant increases in sales of plain milk and untaxed fruit, vegetable, and tea drinks, as well as a significant decline in untaxed diet drinks. Prior evidence suggests that when individuals substitute beverages in the wake of increased SSB prices, they are likely to choose water or diet soft drinks or fruit drinks [33], of which only fruit drinks would add calories. Our point-of-sale data are consistent with regard to increases in sales of water, and possibly fruit drinks, but not diet drinks. Since the point-of-sale data do not show that sales of these “other” untaxed beverages rose meaningfully, perhaps there was an increase in the intake of these beverages at home (prepared from fruit and plain yogurts or milks) or at food-service locations. It is unclear whether consumption changes can be attributed to the tax, and we do not want to speculate since the self-reported beverage intake component of our study did not sample non-Berkeley residents, so we are unable to tell if the increase in the self-reported intake of “other” untaxed beverages was a secular trend or specific to Berkeley. Nonetheless, our results are consistent with Falbe et al.’s findings of small increases for frequency of SSB intake in control communities, no change in location of SSB shopping, and an increase in frequency of water intake in Berkeley [30].

In this comparatively low-SSB-consuming city, the city’s tax revenue over the first year of the SSB tax was $1,416,973 (approximately $12 per capita) [34], roughly four times the 2015 per capita amount in the federal Prevention and Public Health Fund. Proceeds are being used for child nutrition and community health programs [35]. This suggests that SSB taxes can provide significant revenue for prevention or other societal goals.

### Limitations

This observational study cannot establish causal links between the SSB tax implementation and changes in measured outcomes, nor did it assess health outcomes. It cannot distinguish the longer-term effects of education and intensive media debate on SSBs in the communities surrounding the San Francisco Bay as a result of tax and other preexisting campaigns in both Berkeley and San Francisco in 2014, although, in contrast to Falbe et al.’s work, baseline store price survey and consumption data were collected after those campaigns but before tax implementation, mitigating this issue somewhat. Our selection of comparison sites used cities that had been exposed to similar educational campaigns and to the Bay Area tax media campaigns (both for and against), so that the difference-in-difference analysis more purely reflects the effects of the tax itself. The alternative of a more distant control would have better captured the combined effects of the campaigns and the tax itself.

This study also cannot clarify whether distributors, retailers, and/or consumers altered their behaviors in anticipation of the SSB tax or to what degree changes in these various parties’ behaviors were associated with changes in prices and sales. The 26-store survey sample was less representative of small and independent stores than of larger groceries. Analysis of consumption was limited by the small effect size in relation to high standard error and Berkeley’s low baseline consumption, leading to an underpowered sample, and by the absence of a comparison community, suggesting the need for a larger, controlled sample, optimally with higher SSB consumption, more reflective of national consumption patterns. Obtaining such a sample in Berkeley proved unfeasible using the random digit dialing approach and available resources in the time-sensitive 6-wk window between passage of the tax and the original implementation date of 1 January 2015, when baseline survey data were collected.

Despite the large number of transactions, while many grocers were invited, scanner data were limited to two chains of large supermarkets and are not generalizable to all stores or store types. Independently owned small corner stores, in particular, are very different, did not exhibit price changes in our data, and may not have reliable records on their sales. Consequently, Berkeley consumers may have shifted their SSB purchases to independent stores, but our data are unable to determine this. The differential pass-through also warrants further investigation. However, in separate descriptive analyses of food purchases for the Bay Area from the 2014 Nielsen Homescan data [36,37], about 50% of the volume of beverages purchased is from chain groceries (with ≥10 locations nationwide), and only about 2% from independent stores (<10 locations nationally) (per our own calculations) [36]. For this reason, chain groceries likely constitute the most significant consumer SSB purchasing setting.

Strengths of this study include an intimate understanding of the local implementation process, the ability to sample large and small stores and chains, and the large volume of transactions studied. These strengths allow us to begin learning where and to what degree the tax was implemented as well as to observe changes in prices, volume sold, and store revenue.

### Conclusions

These findings suggest that implementing a SSB excise tax was feasible and SSB sales fell concomitantly, while the tax captured revenue for obesity prevention and other societal goals. Whether observed changes in sales were related to enactment of the tax or other local activities cannot be definitively determined due to the observational design. However, the observation of price increases for SSBs in two distinct data sources, the timing of those increases, and the patterns of change in taxed and untaxed beverage sales suggest that the observed changes may be attributable to the tax. Assessment of newly approved SSB taxes in a number of other cities/counties in the US at 1–2¢/oz will be important, and associations of taxation with substitutions in beverage sales and intake should be further assessed in settings with more typical consumption and using larger samples.

## Supporting information

S1 Fig Point-of-sale mean monthly unadjusted sales (ounces/transaction) of beverages in Berkeley versus non-Berkeley stores. (DOCX)

S1 STROBE STROBE checklist for observational studies. (DOCX)

S1 Table Panel of 70 beverage items by beverage and taxation categories collected in the store price surveys conducted in December 2014, June 2015, and March 2016. (DOCX)

S2 Table Number of prices collected across all stores for the standard panel of 70 beverages in the store price surveys, by store and beverage type. (DOCX)

S3 Table Mean (95% CI) store price survey change in beverage prices (cents/ounce) by store type in Berkeley that were collected in all three rounds. (DOCX)

S4 Table Mean (standard error) store price survey change in beverage prices (cents/ounce) by store type in Berkeley based on paired comparisons. (DOCX)

S5 Table Neighborhood characteristics of Berkeley and non-Berkeley grocery stores providing scanner data. American Community Survey 5-y estimates (2009–2013).
(DOCX)

S6 Table Number of barcode scans, unique barcodes, and transactions included in the point-of-sale study. (DOCX)

S7 Table Beverages included and excluded from point-of-sale study. (DOCX)

S8 Table Point-of-sale mean (95% CI) sales-unweighted differences in beverage prices (cents/ounce) in Berkeley versus non-Berkeley stores by beverage group from fixed effects models. (DOCX)

S9 Table Point-of-sale mean (95% CI) sales-weighted differences in beverage prices (cents/ounce) in Berkeley versus non-Berkeley stores by beverage group from fixed effects models. (DOCX)

S10 Table Point-of-sale model adjusted monthly counterfactuals and observed sales of beverages in Berkeley versus non-Berkeley stores, and mean absolute (ounces/transaction) and relative (percent of counterfactual) differences. CF, counterfactual; NB, non-Berkeley.
(DOCX)

S11 Table Point-of-sale model adjusted counterfactuals and observed sales of untaxed beverage categories in Berkeley versus non-Berkeley stores, and mean absolute (ounces/transaction) and relative (percent of counterfactual) differences. CF, counterfactual; NB, non-Berkeley.
(DOCX)

S12 Table Weighted frequency of reported consumption rates of taxed and untaxed beverages by subcategory pre- and post-tax, Berkeley, California, 2014–2015. (DOCX)

S13 Table Descriptive statistics of Berkeley sample in 2014 and 2015 compared to adult sample (age 18 y and older) in the National Health and Nutrition Examination Survey, 2011–2012. (DOCX)

S1 Text Details on the store price survey sample design, data collection procedures, and price analysis. (DOCX)

S2 Text Details on the point-of-sale data and price, volume sold, and store revenue analyses. (DOCX)

S3 Text Point-of-sale price fixed effects models and predictions. (DOCX)

S4 Text Point-of-sale volume sold difference-in-difference models and predicted outcomes. (DOCX)

S5 Text Details on the dietary and shopping behavior survey. (DOCX)

S6 Text Usual intake modeling approach. (DOCX)

S7 Text References for supporting information. (DOCX)

## Abbreviations

CPI: Consumer Price Index
SSB: sugar-sweetened beverage
