# Supplementary material for: Changes in prices, sales, consumer spending, and beverage consumption one year after a tax on sugar-sweetened beverages in Berkeley, California, US: A before-and-after study
Source: PLoS Med. 2017 Apr 18;14(4):e1002283. doi: 10.1371/journal.pmed.1002283 (PMC5395172; doi:10.1371/journal.pmed.1002283)
Supplement: S1 STROBE — (DOCX) [file pmed.1002283.s002.docx]

**S1 STROBE. STROBE Checklist for Observational Studies**

|  | | Item # | | Recommendation *(Location in submission)* | |  |
| --- | --- | --- | --- | --- | --- | --- |
| **Title and abstract** | | 1 | | (*a*) Indicate the study’s design with a commonly used term in the title or the abstract ***(Title “before and after study” and Abstract’s first sentence)*** | |  |
|  |  |  |  | (*b*) Provide in the abstract an informative and balanced summary of what was done and what was found ***(Abstract)*** | |  |
| Introduction | | | | | |  |
| Background/ rationale | | 2 | | Explain the scientific background and rationale for the investigation being reported ***(Introduction paragraph 1-2)*** | |  |
| Objectives | | 3 | | State specific objectives, including any prespecified hypotheses ***(Introduction paragraph 3)*** | |  |
| Methods | | | | | |  |
| Study design | | 4 | | Present key elements of study design early in the paper ***(Methods paragraph 1)*** | |  |
| Setting | | 5 | | Describe the setting, locations, and relevant dates, including periods of recruitment, exposure, follow-up, and data collection ***(Methods, Figure 1 and paragraph 2)*** | |  |
| Participants | | 6 | | *Cross-sectional study*—Give the eligibility criteria, and the sources and methods of selection of participants ***(Methods paragraph 3-6; S1 Text, S2 Text and S5 Text)*** | |  |
| Variables | | 7 | | Clearly define all outcomes, exposures, predictors, potential confounders, and effect modifiers. Give diagnostic criteria, if applicable ***(Analytical approaches paragraph 1-5, S1 Text, S2 Text, S3 Text, S4 Text, S5 Text and S6 Text)*** | |  |
| Data sources/ measurement | | 8* | | For each variable of interest, give sources of data and details of methods of assessment (measurement). Describe comparability of assessment methods if there is more than one group ***(Methods paragraph 3-6; S1 Text, S1 Table, S2 Table, S2 Text, S5 Table, S6 Table, S7 Table, and S5 Text)*** | |  |
| Bias | | 9 | | Describe any efforts to address potential sources of bias ***(Analytical approaches paragraph 1-5, S1 Text, S2 Text, S3 Text, S4 Text, S5 Text, S6 Text)*** | |  |
| Study size | | 10 | | Explain how the study size was arrived at ***(Methods paragraph 3-6; S1 Text, S2 Text and S5 Text)*** | |  |
| Quantitative variables | | 11 | | Explain how quantitative variables were handled in the analyses. If applicable, describe which groupings were chosen and why ***(Analytical approaches paragraph 1-5, S1 Text, S2 Text, S3 Text, S4 Text, S5 Text, S6 Text)*** | |  |
| Statistical methods | | 12 | | (*a*) Describe all statistical methods, including those used to control for confounding ***(Analytical approaches paragraph 1-5, S3 Text, S4 Text, S6 Text)*** | |  |
|  |  |  |  | (*b*) Describe any methods used to examine subgroups and interactions ***(Analytical approaches paragraph 1-5, S3 Text, S4 Text, and S6 Text)*** | |  |
|  |  |  |  | (*c*) Explain how missing data were addressed ***(Analytical approaches paragraph 1-5, S3 Text, S4 Text, and S6 Text)*** | |  |
|  |  |  |  | (*d*) Describe any sensitivity analyses ***(NA)*** | |  |
| Results | | | | | | |
| Participants | 13* | | (a) Report numbers of individuals at each stage of study—eg numbers potentially eligible, examined for eligibility, confirmed eligible, included in the study, completing follow-up, and analysed ***(Results - Usual intake of beverages from self-reports paragraph 1, S5 Text)*** | |  |  |
|  |  |  | (b) Give reasons for non-participation at each stage ***(S1 Text, S2 Text and S5 Text)*** | |  |  |
|  |  |  | (c) Consider use of a flow diagram ***(Not included, but described in S1 Text, S2 Text and S5 Text)*** | |  |  |
| Descriptive data | 14* | | (a) Give characteristics of study participants (eg demographic, clinical, social) and information on exposures and potential confounders ***(S1 Table, S5 Table, S1 Fig, S12 Table, and S13 Table)*** | |  |  |
|  |  |  | (b) Indicate number of participants with missing data for each variable of interest ***(Given the complexity of this study, we elected not to do this, but describe how we ended up with our final analytical samples in*** ***S1 Text, S2 Text and S5 Text)*** | |  |  |
| Outcome data | 15* | | *Cross-sectional study—*Report numbers of outcome events or summary measures ***(Fig 2, S3 Table, Fig 3, S8 Table, Fig 4, Table 1 and S12 Table)*** | |  |  |
| Main results | 16 | | (*a*) Give unadjusted estimates and, if applicable, confounder-adjusted estimates and their precision (eg, 95% confidence interval). Make clear which confounders were adjusted for and why they were included ***(Results paragraph 1-7, Fig 3-5, Table 1, S3 Text, S4 Text, S6 Text, S3 Table, S8 Table, S12 Table, S13 Table S1 Fig)*** | |  |  |
|  |  |  | (*b*) Report category boundaries when continuous variables were categorized **(*NA)*** | |  |  |
|  |  |  | (*c*) If relevant, consider translating estimates of relative risk into absolute risk for a meaningful time period ***(NA)*** | |  |  |
| Other analyses | 17 | | Report other analyses done—eg analyses of subgroups and interactions, and sensitivity analyses ***(S4 Table, S9 Table, S10 Table, S11 Table)*** | |  |  |
| Discussion | | | | |  |  |
| Key results | 18 | | Summarise key results with reference to study objectives ***(Discussion paragraph 1)*** | |  |  |
| Limitations | 19 | | Discuss limitations of the study, taking into account sources of potential bias or imprecision. Discuss both direction and magnitude of any potential bias ***(Limitations)*** | |  |  |
| Interpretation | 20 | | Give a cautious overall interpretation of results considering objectives, limitations, multiplicity of analyses, results from similar studies, and other relevant evidence ***(Discussion paragraph 2-5, and Conclusions)*** | |  |  |
| Generalisability | 21 | | Discuss the generalisability (external validity) of the study results ***(Limitations paragraph 3 and 4, and Conclusions )*** | |  |  |
| Other information | | | | |  |  |
|  | 22 | | Give the source of funding and the role of the funders for the present study and, if applicable, for the original study on which the present article is based ***(Financial Disclosure statement)*** | |  |  |
