# Supplementary material for: Changes in prices, sales, consumer spending, and beverage consumption one year after a tax on sugar-sweetened beverages in Berkeley, California, US: A before-and-after study
Source: PLoS Med. 2017 Apr 18;14(4):e1002283. doi: 10.1371/journal.pmed.1002283 (PMC5395172; doi:10.1371/journal.pmed.1002283)
Supplement: S1 Table — (DOCX) [file pmed.1002283.s003.docx]

S1 Table Panel of 70 beverage items by Beverage and Taxation Categories collected in the Store Price Surveys conducted in December 2014, June 2015 and March 2016

| **Beverage Category** | **Taxed Beverages** | | **Untaxed Beverages** | |
| --- | --- | --- | --- | --- |
|  | **Product Name** | **Size** | **Product Name** | **Size** |
| **Soda** | Coke | 12 oz ^ | Diet Coke | 12 oz ^ |
|  | Coke | 20 oz ^ | Diet Coke | 20 oz ^ |
|  | Coke | 2 liter ^ | Diet Coke | 2 liter ^ |
|  |  |  | Coke Zero | 20 oz ^ |
|  |  |  | Coke Zero | 2 liter ^ |
|  | Dr. Pepper | 12 oz ^ | Diet Dr. Pepper | 12 oz ^ |
|  | Dr. Pepper | 2 liter ^ | Diet Dr. Pepper | 2 liter ^ |
|  | Fanta Orange | 16 oz | Diet Fanta Orange | 16 oz |
|  | Jarritos Mandarina | 12.5 oz ^ | Jarritos Mandarina Light | 12.5 oz |
|  | Mountain Dew | 12 oz ^ | Diet Mountain Dew | 12 oz |
|  | Mountain Dew | 20 oz ^ | Diet Mountain Dew | 20 oz ^ |
|  | Mountain Dew | 2 liter ^ | Diet Mountain Dew | 2 liter ^ |
|  | Pepsi | 12 oz ^ | Diet Pepsi | 12 oz ^ |
|  | Pepsi | 20 oz ^ | Diet Pepsi | 20 oz ^ |
|  | Pepsi | 2 liter ^ | Diet Pepsi | 2 liter ^ |
|  | Sprite | 12 oz ^ | Diet Sprite | 12 oz |
|  | Sprite | 20 oz ^ | Diet Sprite | 20 oz |
|  | Sprite | 2 liter ^ | Diet Sprite | 2 liter |
|  | Sunkist orange | 20 oz ^ |  |  |
| **Energy Drinks** | Monster Energy | 16 oz ^ | Low Carb Monster Energy | 16 oz ^ |
|  | Red Bull | 8.4 oz ^ | Red Bull Sugarfree | 8.4 oz ^ |
|  | Rockstar | 16 oz ^ | Rockstar Pure Zero | 16 oz ^ |
| **Juice drinks** | Capri Sun Fruit Punch | 6 oz ^ |  |  |
|  | Sunny Delight Tangy Original | 11.3 oz |  |  |
|  | Sunny Delight Tangy Original | 2 liters |  |  |
| **Sports Drinks** | Gatorade Fruit Punch | 28 oz ^ | Propel Flavored Water Grape | 24 oz |
| **Tea** | Arizona Iced Tea Lemon | 23 oz ^ |  |  |
|  | Arizona Iced Tea Green | 23 oz ^ | Diet Arizona Green Iced Tea | 23 oz |
|  | Snapple Lemon | 16.9 oz ^ | Snapple Diet Lemon | 16.9 oz ^ |
|  | Synergy Kombucha Gingerade | 16 oz ^ |  |  |
| **100% Juice** |  |  | Berkeley Farms Orange Juice | 1.89 liters |
|  |  |  | Minute Maid Orange Juice | 15.2 oz ^ |
|  |  |  | Tropicana Orange Juice | 12 oz ^ |
|  |  |  | Tropicana Orange Juice | 59 oz ^ |
|  |  |  | VitaCoco Pure Coconut Water | 16.9 oz ^ |
| **Milk** |  |  | Berkeley Farms Whole | 1/2 gallon ^ |
|  |  |  | Berkeley Farms Whole | Gallon ^ |
|  |  |  | Horizon Whole | 1/2 gallon ^ |
|  |  |  | Horizon Whole | Gallon |
|  |  |  | Berkeley Farms 1% | 1/2 gallon ^ |
|  |  |  | Berkeley Farms 1% | Gallon ^ |
|  |  |  | Horizon 1% | 1/2 gallon |
|  |  |  | Horizon 1% | Gallon |
| **Water** |  |  | Aquafina | 1 liter ^ |
|  |  |  | Arrowhead | 1 liter ^ |
|  |  |  | Crystal Geyser | 23.6 oz ^ |
|  |  |  | Crystal Geyser | 1 liter ^ |
|  |  |  | Dasani | 20 oz ^ |

Note: ^ Beverage price collected during all three rounds of the study in one or more stores. 55 of the 70 beverage types were collected at all three rounds during the study (December 2014, June 2015, and March 2016).
