# Supplementary material for: Changes in prices, sales, consumer spending, and beverage consumption one year after a tax on sugar-sweetened beverages in Berkeley, California, US: A before-and-after study
Source: PLoS Med. 2017 Apr 18;14(4):e1002283. doi: 10.1371/journal.pmed.1002283 (PMC5395172; doi:10.1371/journal.pmed.1002283)
Supplement: S2 Table — (DOCX) [file pmed.1002283.s004.docx]

**S2 Table Number of prices collected across all stores for the standard panel of 70 beverages in the Store Price Surveys, by store and beverage type**

|  | **December 2014** | | **June 2015** | | **March 2016** | |
| --- | --- | --- | --- | --- | --- | --- |
| **All Stores (N=26)** | **Taxed** | **Untaxed** | **Taxed** | **Untaxed** | **Taxed** | **Untaxed** |
| Soda | 173 | 100 | 184 | 133 | 165 | 104 |
| Energy drinks | 51 | 48 | 55 | 53 | 48 | 35 |
| Juice drinks | 4 |  | 5 |  | 2 |  |
| Sport drinks | 9 | 2 | 15 | 2 | 10 | 0 |
| Tea | 44 | 5 | 39 | 5 | 38 | 8 |
| 100% Juice |  | 43 |  | 38 |  | 34 |
| Milk |  | 45 |  | 45 |  | 41 |
| Water |  | 36 |  | 28 |  | 26 |
| **Large chain supermarkets (N=6)** |  |  |  |  |  |  |
| Soda | 44 | 27 | 45 | 34 | 39 | 34 |
| Energy drinks | 11 | 11 | 13 | 13 | 11 | 11 |
| Juice drinks | 0 |  | 0 |  | 0 |  |
| Sport drinks | 1 | 2 | 1 | 1 | 1 | 2 |
| Tea | 13 | 1 | 10 | 2 | 9 | 1 |
| 100% Juice |  | 11 |  | 14 |  | 11 |
| Milk |  | 13 |  | 17 |  | 13 |
| Water |  | 10 |  | 9 |  | 10 |
| **Small chain supermarkets (N=2) or chain gas stations (N=2)** |  |  |  |  |  |  |
| Soda | 20 | 13 | 20 | 17 | 21 | 15 |
| Energy drinks | 9 | 9 | 9 | 7 | 9 | 7 |
| Juice drinks | 0 |  | 0 |  | 0 |  |
| Sport drinks | 2 | 1 | 3 | 0 | 3 | 0 |
| Tea | 5 | 0 | 5 | 0 | 6 | 2 |
| 100% Juice |  | 6 |  | 8 |  | 8 |
| Milk |  | 6 |  | 9 |  | 3 |
| Water |  | 3 |  | 6 |  | 5 |
| **Pharmacies (N=2)** |  |  |  |  |  |  |
| Soda | 17 | 11 | 16 | 18 | 14 | 11 |
| Energy drinks | 6 | 5 | 5 | 5 | 6 | 6 |
| Juice drinks | 0 |  | 0 |  | 0 |  |
| Sport drinks | 0 | 0 | 2 | 0 | 1 | 0 |
| Tea | 6 | 0 | 5 | 0 | 3 | 1 |
| 100% Juice |  | 5 |  | 7 |  | 6 |
| Milk |  | 4 |  | 5 |  | 4 |
| Water |  | 4 |  | 1 |  | 3 |
| **Independent corner stores (N=13) or independent gas station (N=1)** |  |  |  |  |  |  |
| Soda | 106 | 58 | 114 | 76 | 101 | 53 |
| Energy drinks | 28 | 26 | 31 | 31 | 25 | 15 |
| Juice drinks | 4 |  | 5 |  | 2 |  |
| Sport drinks | 7 | 0 | 10 | 1 | 6 | 0 |
| Tea | 23 | 4 | 20 | 1 | 21 | 3 |
| 100% Juice |  | 25 |  | 14 |  | 14 |
| Milk |  | 27 |  | 19 |  | 25 |
| Water |  | 21 |  | 15 |  | 12 |

Note: Gray cells indicate that there were no items included in the standard beverage panel (see S1 Table).
