# Supplementary material for: Changes in prices, sales, consumer spending, and beverage consumption one year after a tax on sugar-sweetened beverages in Berkeley, California, US: A before-and-after study
Source: PLoS Med. 2017 Apr 18;14(4):e1002283. doi: 10.1371/journal.pmed.1002283 (PMC5395172; doi:10.1371/journal.pmed.1002283)
Supplement: S3 Table — (DOCX) [file pmed.1002283.s005.docx]

S3 Table Store Price Survey change in beverage prices (cents/oz) by store types in Berkeley that were collected in all three rounds, mean and 95% confidence intervals

| **Large supermarkets (n=6)** | **Taxed Beverage price**  **(36 sets)** | | | **Untaxed Beverage price**  **(36 sets)** | | | **Taxed – Untaxed Difference** | | |
| --- | --- | --- | --- | --- | --- | --- | --- | --- | --- |
|  | **cents/oz** | **95% CI** | | **cents/oz** | **95% CI** | | **cents/oz** | **95% CI** | |
| Round 1: December 2014 | 15.62 | 10.15 | 21.08 | 11.19 | 7.45 | 14.93 |  |  |  |
| Round 2: June 2015 | 16.93 | 11.29 | 22.57 | 11.48 | 7.64 | 15.32 |  |  |  |
| Round 3: March 2016 | 16.68 | 11.26 | 22.10 | 11.70 | 7.79 | 15.61 |  |  |  |
| *Mean change*  *(March 2016–Dec 2014)* | *1.07**  *(p=0.01)* | *0.22* | *1.91* | *0.51**  *(p=0.01)* | *0.16* | *0.86* | *0.56*  *(p=0.22)* | *-0.35* | *1.46* |
| *Mean change*  *(June 2015–Dec 2014)* | *1.31***  *(p<0.001)* | *0.75* | *1.87* | *0.29*  *(p=0.08)* | *-0.03* | *0.61* | *1.02 ^‡^*  *(p=0.002)* | *0.39* | *1.66* |
| **Small chain supermarkets (n=2) or chain gas stations (n=2)** | **Taxed Beverages price**  **(20 sets)** | | | **Untaxed Beverage price (20 sets)** | | | **Taxed – Untaxed Difference** | | |
|  | **cents/oz** | **95% CI** | | **cents/oz** | **95% CI** | | **cents/oz** | **95% CI** | |
| Round 1: December 2014 | 23.06 | 15.00 | 31.12 | 17.59 | 10.96 | 24.21 |  |  |  |
| Round 2: June 2015 | 25.26 | 16.31 | 34.21 | 18.21 | 11.42 | 25.01 |  |  |  |
| Round 3: March 2016 | 24.37 | 15.82 | 32.92 | 18.47 | 11.46 | 25.48 |  |  |  |
| *Mean change*  *(March 2016–Dec 2014)* | *1.31***  *(p=0.004)* | *0.46* | *2.16* | *0.88***  *(p=0.002)* | *0.39* | *1.38* | *0.43*  *(p=0.37)* | *-0.53* | *1.38* |
| *Mean change*  *(June 2015–Dec 2014)* | *2.20**  *(p=0.03)* | *0.27* | *4.14* | *0.63**  *(p=0.04)* | *0.02* | *1.23* | *1.58*  *(p=0.12)* | *-0.43* | *3.58* |
| **Pharmacies (n=2)** | **Taxed Beverage price**  **(18 sets)** | | | **Untaxed Beverage price (18 sets)** | | | **Taxed – Untaxed Difference** | | |
|  | **cents/oz** | **95% CI** | | **cents/oz** | **95% CI** | | **cents/oz** | **95% CI** | |
| Round 1: December 2014 | 18.18 | 9.16 | 27.21 | 15.20 | 9.47 | 20.92 |  |  |  |
| Round 2: June 2015 | 19.08 | 9.58 | 28.58 | 16.08 | 9.39 | 22.76 |  |  |  |
| Round 3: March 2016 | 18.63 | 9.51 | 27.75 | 15.44 | 9.77 | 21.10 |  |  |  |
| *Mean change*  *(March 2016–Dec 2014)* | *0.45***  *(p=0.03)* | *0.04* | *0.85* | *0.24*  *(p=23)* | *-0.17* | *0.65* | *0.21*  *(p=0.45)* | *-0.34* | *0.76* |
| *Mean change*  *(June 2015–Dec 2014)* | *0.90*  *(p=0.36)* | *-1.10* | *2.89* | *0.88*  *(p=36)* | *-1.11* | *2.87* | *0.02*  *(p=0.99)* | *-2.70* | *2.73* |
| **Independent corner stores (n=13) or independent gas stations (n=1)** | **Taxed Beverage price**  **(103 sets)** | | | **Untaxed Beverage price (62 sets)** | | | **Taxed – Untaxed Difference** | | |
|  | **cents/oz** | **95% CI** | | **cents/oz** | **95% CI** | | **cents/oz** | **95% CI** | |
| Round 1: December 2014 | 15.21 | 12.28 | 18.14 | 13.04 | 9.92 | 16.16 |  |  |  |
| Round 2: June 2015 | 15.12 | 12.26 | 17.99 | 13.01 | 9.98 | 16.03 |  |  |  |
| Round 3: March 2016 | 14.57 | 11.83 | 17.31 | 12.43 | 9.60 | 15.27 |  |  |  |
| *Mean change*  *(March 2016–Dec 2014)* | *-0.64***  *(p=0.004)* | *-1.08* | *-0.21* | *-0.61**  *(p=0.03)* | *-1.15* | *-0.07* | *-0.04*  *(p=0.92)* | *-0.72* | *0.65* |
| *Mean change*  *(June 2015–Dec 2014)* | *-0.09*  *(p=0.62)* | *-0.44* | *0.26* | *-0.03*  *(p=0.85)* | *-0.37* | *0.30* | *-0.06*  *(p=0.82)* | *-0.54* | *0.42* |

Notes: Prices account for inflation over time.

Sets means the number of beverage prices collected at all three rounds for the same beverage item from a particular store. For example, if the price of a 12 ounce can of Pepsi was collected at December 2014, June 2015 and March 2016 at the same store, we would consider that one set.

n= number of stores of each type; ** denotes statistical significant difference between prices in March 2016 compared to earlier round (December 2014 or June 2015) at p<0.01 using paired t-tests. * denote statistical significant difference between prices in March 2016 compared to earlier round (December 2014 or June 2015) at p<0.05 using paired-t-tests.

^‡^ denotes statistical significant difference of price of taxed beverages compared to untaxed beverages at p<0.05 (unpaired t-tests since taxed and untaxed beverage items are different).

Source: PHI Store Price Survey data collected.
