# Supplementary material for: Changes in prices, sales, consumer spending, and beverage consumption one year after a tax on sugar-sweetened beverages in Berkeley, California, US: A before-and-after study
Source: PLoS Med. 2017 Apr 18;14(4):e1002283. doi: 10.1371/journal.pmed.1002283 (PMC5395172; doi:10.1371/journal.pmed.1002283)
Supplement: S4 Table — (DOCX) [file pmed.1002283.s006.docx]

S4 Table Store Price Survey change in beverage prices (cents/oz) beverages by store types in Berkeley based on paired comparisons, mean (SE)

| **Large supermarkets (n=6)** | **Taxed Beverages** | | | **Untaxed Beverages** | | | **Taxed – Untaxed Difference** |
| --- | --- | --- | --- | --- | --- | --- | --- |
|  | **n** | **cents/oz** | **SE** | **n** | **cents/oz** | **SE** | **cents/oz** |
| Round 1: December 2014 | 48 | 15.01 | 2.29 | 51 | 10.79 | 1.45 | 4.22 |
| Round 2: June 2015 | 53 | 15.91 | 2.25 | 62 | 11.50 | 1.38 | 4.41 |
| Round 3: March 2016 | 45 | 16.36 | 2.24 | 55 | 11.10 | 1.39 | 5.26 |
| *Mean change*  *(March 2016–Dec 2014)* | *39 pairs* | *1.10*** | *0.39* | *39 pairs* | *0.66** | *0.22* | *0.44* |
| *Mean change*  *(June 2015–Dec 2014)* | *42 pairs* | *1.33*** | *0.26* | *43 pairs* | *0.17* | *0.18* | *1.16* |
| **Small chain supermarkets (n=2) or chain gas stations (n=2)** | **Taxed Beverages** | | | **Untaxed Beverages** | | | **Taxed – Untaxed Difference** |
|  | **n** | **cents/oz** | **SE** | **n** | **cents/oz** | **SE** | **cents/oz** |
| Round 1: December 2014 | 36 | 15.94 | 2.53 | 38 | 14.57 | 2.15 | 1.37 |
| Round 2: June 2015 | 37 | 19.08 | 2.68 | 47 | 12.68 | 1.70 | 6.40 |
| Round 3: March 2016 | 39 | 17.51 | 2.45 | 40 | 13.94 | 1.93 | 3.57 |
| *Mean change*  *(March 2016–Dec 2014)* | *26 pairs* | *1.11** | *0.38* | *26 pairs* | *0.90*** | *0.24* | *0.20* |
| *Mean change*  *(June 2015–Dec 2014)* | *24 pairs* | *2.09** | *0.77* | *26 pairs* | *0.53** | *0.23* | *1.56* |
| **Pharmacies (n=2)** | **Taxed Beverages** | | | **Untaxed Beverages** | | | **Taxed – Untaxed Difference** |
|  | **n** | **cents/oz** | **SE** | **n** | **cents/oz** | **SE** | **cents/oz** |
| Round 1: December 2014 | 29 | 14.99 | 2.97 | 29 | 12.27 | 2.00 | 2.73 |
| Round 2: June 2015 | 28 | 14.37 | 3.13 | 36 | 12.44 | 1.99 | 1.93 |
| Round 3: March 2016 | 24 | 16.90 | 3.40 | 31 | 13.83 | 2.13 | 3.08 |
| *Mean change*  *(March 2016–Dec 2014)* | *20 pairs* | *0.46*** | *0.17* | *21 pairs* | *0.25* | *0.17* | *0.21* |
| *Mean change*  *(June 2015–Dec 2014)* | *24 pairs* | *0.67* | *0.71* | *25 pairs* | *0.61* | *0.68* | *0.06* |
| **Independent corner stores (n=13) or independent gas stations (n=1)** | **Taxed Beverages** | | | **Untaxed Beverages** | | | **Taxed – Untaxed Difference** |
|  | **n** | **cents/oz** | **SE** | **n** | **cents/oz** | **SE** | **cents/oz** |
| Round 1: December 2014 | 168 | 14.07 | 1.09 | 161 | 12.45 | 0.92 | 1.63 |
| Round 2: June 2015 | 180 | 14.34 | 1.10 | 159 | 13.15 | 0.97 | 1.18 |
| Round 3: March 2016 | 155 | 14.46 | 1.15 | 122 | 10.49 | 0.87 | 3.97 |
| *Mean change*  *(March 2016–Dec 2014)* | *120 pairs* | *-0.67*** | *0.20* | *81 pairs* | *-0.41* | *0.25* | *-0.26* |
| *Mean change*  *(June 2015–Dec 2014)* | *137 pairs* | *-0.25* | *0.17* | *102 pairs* | *-0.30* | *0.20* | *0.05* |

Notes: Prices account for inflation over time.

Pairs means the number of beverage prices collected at two rounds for the same beverage item from a particular store. For example, if the price of a 12 ounce can of Pepsi was collected at December 2014 and June 2015 at the same store, we would consider that one pair.

n= number of prices records across stores. Comparisons are only among matched products over time. ** denotes statistical significant difference between prices in March 2016 compared to earlier round (December 2014 or June 2015) at p<0.01 using paired t-tests. * denote statistical significant difference between prices in March 2016 compared to earlier round (December 2014 or June 2015) at p<0.05 using paired-t-tests.

Source: PHI Store Price Survey data collected.
