# Supplementary material for: Changes in prices, sales, consumer spending, and beverage consumption one year after a tax on sugar-sweetened beverages in Berkeley, California, US: A before-and-after study
Source: PLoS Med. 2017 Apr 18;14(4):e1002283. doi: 10.1371/journal.pmed.1002283 (PMC5395172; doi:10.1371/journal.pmed.1002283)
Supplement: S5 Table — American Community Survey 5-y estimates (2009–2013). (DOCX) [file pmed.1002283.s007.docx]

S5 Table Neighborhood characteristics of Berkeley and Non-Berkeley grocery stores providing scanner data. American Community Survey 5-Year Estimates (2009-2013)

| **Neighborhood Characteristics** | **Berkeley neighborhoods (n=3)** | **Non-Berkeley neighborhoods (n=6)** |
| --- | --- | --- |
| Age category (%) |  |  |
| Less than 18 years | 17.3% | 17.9% |
| 65+ years | 14.3% | 15.5% |
| Race (%) |  |  |
| White | 63.9% | 60.7% |
| African American or black | 11.4% | 9.5% |
| Asian | 13.7% | 17.8% |
| Other (includes those who identify with 2 or more races) | 11.0% | 12.0% |
| Hispanic or Latino ethnicity (%) | 12.5% | 13.5% |
| Foreign-born (%) | 21.5% | 26.0% |
| Median household income (in 2013 inflation-adjusted dollars) | $86,462 | $84,411 |
| Percent with SNAP benefits in past 12 months | 4.3% | 2.7% |
| <100% Federal Poverty Level (FPL) during past 12 months | 14.4% | 11.7% |
| Bachelor's degree or higher (among those ≥25y) | 71.0% | 50.8% |
| Source: US Census Bureau, 2009-2013 American Community Survey, [www.factfinder.census.gov](http://www.factfinder.census.gov). Note that this data is derived at a more granular level than the US Census Bureau data on Berkeley City. Not all neighborhoods in Berkeley City are included in this table. | | |
