# Supplementary material for: Changes in prices, sales, consumer spending, and beverage consumption one year after a tax on sugar-sweetened beverages in Berkeley, California, US: A before-and-after study
Source: PLoS Med. 2017 Apr 18;14(4):e1002283. doi: 10.1371/journal.pmed.1002283 (PMC5395172; doi:10.1371/journal.pmed.1002283)
Supplement: S6 Table — (DOCX) [file pmed.1002283.s008.docx]

S6 Table Number of barcode-scans, unique barcodes and transactions included in the Point-of-sales study

| **# Barcode-scans** |  |
| --- | --- |
| Total UPC scans (beverages, food and non-foods) | 118,792,416 |
| Total UPC beverage-type scans | 16,155,351 |
| Total UPC beverage scans of products included in this study  (% of beverage scans) | 10,777,153  (66.7%) |
| **# Unique Barcodes** |  |
| Beverage, foods and non-food products | 123,713 |
| Beverage-type products | 16,769 |
| Beverage products included in this study  (% of beverage products) | 5,631  (33.6%) |
| **# Transactions that included:** |  |
| Beverage, foods and non-food products | 15,540,933 |
| Beverage-type products | 8,179,804 |
| Beverage products included in this study  (% of beverage products) | 6,078,738  (74.3%) |
| Notes: Barcode-scans refer to each time a barcode is scanned at checkout. Transactions or checkout episodes refer to each time a shopper checks out—a transaction may involve many barcode-scans. | |
