# Supplementary material for: Changes in prices, sales, consumer spending, and beverage consumption one year after a tax on sugar-sweetened beverages in Berkeley, California, US: A before-and-after study
Source: PLoS Med. 2017 Apr 18;14(4):e1002283. doi: 10.1371/journal.pmed.1002283 (PMC5395172; doi:10.1371/journal.pmed.1002283)
Supplement: S7 Table — (DOCX) [file pmed.1002283.s009.docx]

S7 Table Beverages included and excluded from Point-of-sales study

| **Beverage Category** | **Beverages included and examples** |
| --- | --- |
| Soft drinks & energy drinks | Soft drinks (e.g., Coca-Cola Classic, Sprite) |
|  | Energy drinks (e.g., Red Bull) |
| Fruit, vegetable or tea drinks | Fruit drinks, sports drinks (e.g., Capri Sun Fruit Punch, Gatorade G2) |
|  | Ready-to-drink teas without milk/creamer (e.g., Gold Peak Sweet Tea, Snapple Iced Tea) |
|  | Fruit juices (e.g., Ocean Spray Cranberry Juice Cocktail, Welch’s 100% Grape Juice) |
|  | Vegetable Juices (e.g., Bolthouse Daily Greens, Odwalla Carrot Juice, V-8 Vegetable Juice) |
| Flavored milk or substitute beverages | Milk, sweetened or flavored (e.g., Horizon Strawberry Milk, Nesquik Chocolate Milk) |
|  | Cocoa and sweetened beverages containing milk/creamers (e.g., Starbucks Mocha Frappuccino) |
|  | Milk substitutes and milk-based beverages (e.g., Rice Dream Original, Silk Soymilk) |
|  | Yogurt drinks/kefir (e.g., Dannon Danimals, Lifeway Kefir) |
| Plain waters* | Plain waters (e.g., sparkling water, still bottled waters, club soda) |
| Plain milks* | Milk, plain (e.g., 1%, 2% or whole milk) |
| Notes:   *denotes all products in this beverage group are untaxed; all other beverage groups included in this study had both taxed and untaxed items.  ^†^We exclude non-ready-to-drink items such as ground/ whole bean coffee; tea bags/loose tea; liquid and powder concentrates/mixes because the taxation status of products that required reconstitution were initially unclear to stores. Moreover, these beverages are sold in weight units (g) rather than liquid volume units (fl oz or mL). We also excluded alcohol (beer, wine, liquor, mixed alcoholic beverages), meal replacements, medical/dietary supplements, infant formula, any product added to another beverage (e.g., syrups, creamers). Lastly, we excluded sales of ready-to-drink fountain or hot drinks with no objective size information (e.g., descriptors are “small/medium/large”) as we were unable to appropriate use these data. | |
