# Supplementary material for: Changes in prices, sales, consumer spending, and beverage consumption one year after a tax on sugar-sweetened beverages in Berkeley, California, US: A before-and-after study
Source: PLoS Med. 2017 Apr 18;14(4):e1002283. doi: 10.1371/journal.pmed.1002283 (PMC5395172; doi:10.1371/journal.pmed.1002283)
Supplement: S8 Table — (DOCX) [file pmed.1002283.s010.docx]

S8 Table Point-of-sales mean sales-unweighted differences in beverage prices (cents/oz) in Berkeley vs non-Berkeley stores by beverage groups from Fixed Effects models, mean (95% confidence intervals)

| **All beverages included in study** | **Taxed Beverages (N=116,728)** | | | **Untaxed Beverages (N=164,124)** | | |
| --- | --- | --- | --- | --- | --- | --- |
|  | **Berkeley** | **Non-Berkeley** | **Diff (B-NB)** | **Berkeley** | **Non-Berkeley** | **Diff (B-NB)** |
| Pre-tax period 1: March-Dec 2013 | 13.05 | 12.99 | 0.07 | 11.81 | 11.78 | 0.04* |
|  | (12.97 , 13.13) | (12.91, 13.06) | (-0.08, 0.21) | (11.78, 11.85) | (11.75, 11.80) | (0.001, 0.07) |
| Pre-tax period 2: March-Dec 2014 | 13.33 | 13.19 | 0.14 * | 11.97 | 11.97 | 0.00 |
|  | (13.27, 13.38) | (13.11, 13.26) | (0.02, 0.26) | (11.93, 12.02) | (11.94, 12.01) | (-0.06, 0.06) |
| Post-tax period:  March-Dec 2015 | 14.16 | 13.35 | 0.81 ** | 12.29 | 12.28 | 0.02 |
|  | (13.94, 14.37) | (13.28, 13.42) | (0.54, 1.08) | (12.22, 12.37) | (12.21, 12.34) | (-0.10, 0.14) |
| *Absolute Difference*  *(Posttax - Pretax2)* | *0.83***  *p=0.00* | *0.16***  *p=0.00* | *0.67***  *p=0.00* | *0.32***  *p=0.00* | *0.30***  *p=0.00* | *0.02*  *p=0.65* |
|  | (0.64, 1.02) | (0.07, 0.25) | (0.46, 0.87) | *(0.28, 0.36)* | *(0.23, 0.37)* | *(-0.06, 0.10)* |
| **Sodas & energy drinks** | **Taxed (N=45,614)** | | | **Untaxed (N=26,796)** | | |
|  | **Berkeley** | **Non-Berkeley** | **Diff (B-NB)** | **Berkeley** | **Non-Berkeley** | **Diff (B-NB)** |
| Pre-tax period 1: March-Dec 2013 | 13.16 | 13.07 | 0.09 | 9.63 | 9.62 | 0.01 |
|  | (13.03, 13.30) | (13.03, 13.11) | (-0.07, 0.25) | (9.53, 9.72) | (9.51, 9.72) | (-0.12, 0.14) |
| Pre-tax period 2: March-Dec 2014 | 13.44 | 13.29 | 0.16* | 9.57 | 9.65 | -0.08 |
|  | (13.34, 13.55) | (13.22, 13.35) | (0.04, 0.28) | (9.41, 9.73) | (9.52, 9.79) | (-0.37, 0.21) |
| Post-tax period:  March-Dec 2015 | 14.70 | 13.45 | 1.25** | 9.83 | 9.67 | 0.16 |
|  | (14.39, 15.00) | (13.34, 13.56) | (0.89, 1.61) | (9.34, 10.33) | (9.56, 9.79) | (-0.44, 0.76) |
| *Absolute Difference*  *(Posttax - Pretax2)* | *1.25***  *p=0.00* | *0.16***  *p=0.003* | *1.09***  *p=0.00* | *0.26*  *p=0.22* | *0.02*  *p=0.41* | *0.24*  *p=0.26* |
|  | *(0.88, 1.62)* | *(0.05, 0.27)* | *(0.71, 1.47)* | *(-0.16, 0.69)* | *(-0.03, 0.08)* | *(-0.18, 0.66)* |
| **Fruit, vegetable or tea drinks** | **Taxed (N=61,262)** | | | **Untaxed (N=79,584)** | | |
|  | **Berkeley** | **Non-Berkeley** | **Diff (B-NB)** | **Berkeley** | **Non-Berkeley** | **Diff (B-NB)** |
| Pre-tax period 1: March-Dec 2013 | 12.53 | 12.50 | 0.04 | 14.02 | 13.96 | 0.07 |
|  | (12.37, 12.69) | (12.39, 12.61) | (-0.21, 0.28) | (13.94, 14.11) | (13.91, 14.00) | (-0.02, 0.15) |
| Pre-tax period 2: March-Dec 2014 | 12.83 | 12.70 | 0.13 | 14.17 | 14.14 | 0.03 |
|  | (12.70, 12.96) | (12.61, 12.79) | (-0.08, 0.34) | (14.09, 14.25) | (14.10, 14.19) | (-0.09, 0.15) |
| Post-tax period:  March-Dec 2015 | 13.41 | 12.87 | 0.54** | 14.60 | 14.64 | -0.04 |
|  | (13.23, 13.58) | (12.79, 12.94) | (0.32, 0.76) | (14.43, 14.76) | (14.53, 14.74) | (-0.28, 0.21) |
| *Absolute Difference*  *(Posttax - Pretax2)* | *0.58***  *p=0.00* | *0.17***  *p=0.00* | *0.41***  *p=0.00* | *0.43***  *p=0.00* | *0.49***  *p=0.00* | *-0.06*  *p=0.39* |
|  | *(0.44, 0.71)* | *(0.07, 0.27)* | *(0.24, 0.58)* | *(0.30, 0.55)* | *(0.40, 0.58)* | *(-0.21, 0.08)* |
| **Flavored milk or substitute beverages** | **Taxed (N=9,852)** | | | **Untaxed (N= 28,551)** | | |
|  | **Berkeley** | **Non-Berkeley** | **Diff (B-NB)** | **Berkeley** | **Non-Berkeley** | **Diff (B-NB)** |
| Pre-tax period 1: March-Dec 2013 | 15.73 | 15.70 | 0.03 | 12.89 | 12.86 | 0.03 |
|  | (15.63, 15.83) | (15.61, 15.79) | (-0.12, 0.18) | (12.84, 12.94) | (12.82, 12.91) | (-0.06, 0.12) |
| Pre-tax period 2: March-Dec 2014 | 15.83 | 15.84 | -0.01 | 13.16 | 13.16 | 0.01 |
|  | (15.68, 15.98) | (15.76, 15.91) | (-0.19, 0.18) | (13.11, 13.22) | (13.13, 13.19) | (-0.06, 0.07) |
| Post-tax period:  March-Dec 2015 | 16.23 | 15.95 | 0.28* | 13.39 | 13.38 | 0.01 |
|  | (16.03, 16.43) | (15.87, 16.03) | (0.05, 0.52) | (13.34, 13.44) | (13.33, 13.43) | (-0.09, 0.10) |
| *Absolute Difference*  *(Posttax - Pretax2)* | *0.40**  *p=0.01* | *0.11 **  *p=0.04* | *0.29*  *p=0.09* | *0.22***  *p=0.00* | *0.22***  *p=0.00* | *0.001*  *p=0.98* |
|  | *(0.09, 0.71)* | *(0.003, 0.22)* | *(-0.04, 0.62)* | *(0.18, 0.27)* | *(0.17, 0.28)* | *(-0.07, 0.07)* |
|  | | | | | | |
| **S8 Table continued** | | | | | | |
| **Plain waters** | **No taxed plain waters** | | | **Untaxed (N=14,006)** | | |
|  |  |  |  | **Berkeley** | **Non-Berkeley** | **Diff (B-NB)** |
| Pre-tax period 1: March-Dec 2013 |  |  |  | 6.22 | 6.24 | -0.01 |
|  |  |  |  | (6.19, 6.26) | (6.20, 6.27) | (-0.08 , 0.05) |
| Pre-tax period 2: March-Dec 2014 |  |  |  | 6.34 | 6.39 | -0.06 |
|  |  |  |  | (6.30, 6.37) | (6.32, 6.47) | (-0.15, 0.03) |
| Post-tax period:  March-Dec 2015 |  |  |  | 6.51 | 6.51 | 0.00 |
|  |  |  |  | (6.45, 6.56) | (6.46, 6.55) | (-0.08, 0.08) |
| *Absolute Difference*  *(Posttax - Pretax2)* |  |  |  | *0.17***  *p=0.00* | *0.11**  *p=0.03* | *0.06*  *p=0.31* |
|  |  |  |  | *(0.13, 0.20)* | *(0.01 , 0.21)* | *(-0.05, 0.16)* |
| **Plain milks** | **No taxed plain milks** | | | **Untaxed (N=15,187)** | | |
|  |  |  |  | **Berkeley** | **Non-Berkeley** | **Diff (B-NB)** |
| Pre-tax period 1: March-Dec 2013 |  |  |  | 7.19 | 7.20 | -0.01 |
|  |  |  |  | (7.13, 7.25) | (7.16, 7.24) | (-0.06, 0.05) |
| Pre-tax period 2: March-Dec 2014 |  |  |  | 7.61 | 7.62 | 0.00 |
|  |  |  |  | (7.54, 7.69) | (7.57, 7.66) | (-0.12, 0.11) |
| Post-tax period:  March-Dec 2015 |  |  |  | 7.82 | 7.79 | 0.02 |
|  |  |  |  | (7.66, 7.97) | (7.69, 7.89) | (-0.20, 0.25) |
| *Absolute Difference*  *(Posttax - Pretax2)* |  |  |  | *0.20***  *p=0.00* | *0.17***  *p=0.00* | *0.03*  *p=0.69* |
|  |  |  |  | *(0.09, 0.31)* | *(0.09, 0.26)* | *(-0.11, 0.17)* |

Notes: **^‡^** Every barcode is weighted evenly, rather than by their popularity (market share); N is the sample size of # of barcoded beverages sold per month from the nine stores in the POS data. Fixed Effects models account for the month-year (indicator variables), store located in Berkeley, interaction of Berkeley store and month-year, and an indicator variable of under-reported sales data from store in particular month. Prices account for inflation over time.

B=Berkeley; NB=non-Berkeley.

Jan-Feb of every year was excluded in the price comparisons due to the ambiguous period of Jan-Feb 2015 when the tax implementation was unclear since the original implementation date of Jan 1, 2015 was moved to March 1, 2015.

** denotes statistical significant difference between mean prices in the post-tax period (March-Dec 2016) from pre-tax period 2 (March-Dec 2014) at p<0.01, * denotes statistical significant difference between mean prices in the post-tax period (March-Dec 2016) from pre-tax period 2 (March-Dec 2014) at p<0.05.

Source: PHI Point-of-sales (POS) data from chains of large supermarkets in the Bay Area.
