# Supplementary material for: Changes in prices, sales, consumer spending, and beverage consumption one year after a tax on sugar-sweetened beverages in Berkeley, California, US: A before-and-after study
Source: PLoS Med. 2017 Apr 18;14(4):e1002283. doi: 10.1371/journal.pmed.1002283 (PMC5395172; doi:10.1371/journal.pmed.1002283)
Supplement: S9 Table — (DOCX) [file pmed.1002283.s011.docx]

S9 Table Point-of-sales mean sales-weighted^‡^ differences in beverage prices (cents/oz) in Berkeley vs non-Berkeley stores by beverage groups from Fixed Effects models, mean (95% confidence intervals)

| **All beverages included in study** | **Taxed Beverages (N=1,011)** | | | **Untaxed Beverages (N=1,685)** | | |
| --- | --- | --- | --- | --- | --- | --- |
|  | **Berkeley** | **Non-Berkeley** | **Diff (B-NB)** | **Berkeley** | **Non-Berkeley** | **Diff (B-NB)** |
| Pre-tax period 1: March-Dec 2013 | 7.81 | 8.16 | -0.35 | 5.37 | 5.43 | -0.06 |
|  | (7.54, 8.09) | (7.94, 8.37) | (-0.71, 0.02) | (5.24, 5.51) | (5.28, 5.59) | (-0.24, 0.12) |
| Pre-tax period 2: March-Dec 2014 | 8.31 | 8.84 | -0.53** | 5.87 | 5.92 | -0.05 |
|  | (8.12, 8.51) | (8.72, 8.96) | (-0.75, -0.31) | (5.67, 6.08) | (5.82, 6.02) | (-0.35, 0.26) |
| Post-tax period:  March-Dec 2015 | 9.52 | 9.40 | 0.12 | 6.02 | 6.02 | 0.00 |
|  | (9.20, 9.84) | (9.10, 9.69) | (-0.37, 0.62) | (5.62, 6.43) | (5.77, 6.27) | (-0.58, 0.59) |
| *Absolute Difference*  *(Posttax - Pretax2)* | *1.21***  *p=0.00* | *0.56***  *p=0.002* | *0.65***  *p=0.003* | *0.15*  *p=0.20* | *0.10*  *p=0.33* | *0.05*  *p=0.74* |
|  | *(0.96, 1.46)* | *(0.21, 0.90)* | *(0.23, 1.07)* | *(-0.08, 0.38)* | *(-0.10, 0.30)* | *(-0.25, 0.35)* |
| **Sodas & energy drinks** | **Taxed (N=337)** | | | **Untaxed (N=337)** | | |
|  | **Berkeley** | **Non-Berkeley** | **Diff (B-NB)** | **Berkeley** | **Non-Berkeley** | **Diff (B-NB)** |
| Pre-tax period 1: March-Dec 2013 | 7.56 | 7.46 | 0.1 | 4.92 | 5.22 | -0.3 |
|  | (7.07, 8.06) | (7.20, 7.72) | (-0.50, 0.71) | (4.54, 5.29) | (4.91, 5.52) | (-0.94, 0.34) |
| Pre-tax period 2: March-Dec 2014 | 7.77 | 7.79 | -0.03 | 5.1 | 5.42 | -0.32 |
|  | (7.09, 8.45) | (7.38, 8.21) | (-1.04, 0.99) | (4.79, 5.42 | (5.20, 5.63) | (-0.80, 0.17) |
| Post-tax period:  March-Dec 2015 | 9.09 | 7.93 | 1.16** | 5.05 | 5.17 | -0.12 |
|  | (8.55, 9.62) | (7.63, 8.22) | (0.35, 1.97) | (4.69, 5.42) | (4.97, 5.36) | (-0.55, 0.33) |
| *Absolute Difference*  *(Posttax - Pretax2)* | *1.32***  *p=0.00* | *0.13*  *p=0.36* | *1.19***  *p=0.00* | *-0.05*  *p=0.37* | *-0.25 ** p=0.006* | *0.20**  *p=0.05* |
|  | *(1.02, 1.61)* | *(-0.15, 0.42)* | *(0.77, 1.61)* | *(-0.15, 0.06)* | *(-0.43, -0.07)* | *(-0.0004, 0.41)* |
| **Fruit, vegetable and tea drinks** | **Taxed (N=337)** | | | **Untaxed (N=337)** | | |
|  | **Berkeley** | **Non-Berkeley** | **Diff (B-NB)** | **Berkeley** | **Non-Berkeley** | **Diff (B-NB)** |
| Pre-tax period 1: March-Dec 2013 | 7.18 | 7.41 | -0.23 | 8.00 | 7.99 | 0.01 |
|  | (6.59, 7.76) | (6.99, 7.82) | (-0.71, 0.25) | (7.65, 8.35) | (7.82, 8.15) | (-0.42, 0.44) |
| Pre-tax period 2: March-Dec 2014 | 7.93 | 7.99 | -0.06 | 8.79 | 8.59 | 0.2 |
|  | (7.32, 8.53) | (7.71, 8.26) | (-0.94, 0.81) | (8.44, 9.13) | (8.41, 8.76) | (-0.32, 0.72) |
| Post-tax period:  March-Dec 2015 | 8.53 | 8.25 | 0.28 | 8.81 | 8.98 | -0.17 |
|  | (7.38, 9.68) | (7.72, 8.77) | (-1.26, 1.82) | (8.23, 9.40) | (8.62, 9.33) | (-1.04, 0.711) |
| *Absolute Difference*  *(Posttax - Pretax2)* | *0.60 * (p=0.048)* | *0.26*  *p=0.15* | *0.34*  *p=0.34* | *0.03*  *p=0.87* | *0.39** p=0.001* | *-0.36*  *p=0.09* |
|  | *(0.01, 1.20)* | *(-0.10, 0.62)* | *(-0.35, 1.04)* | *(-0.32, 0.38)* | *(0.16, 0.62)* | *(-0.79, 0.06)* |
| **Flavored milk or substitute beverages** | **Taxed (N=337)** | | | **Untaxed (N=337)** | | |
|  | **Berkeley** | **Non-Berkeley** | **Diff (B-NB)** | **Berkeley** | **Non-Berkeley** | **Diff (B-NB)** |
| Pre-tax period 1: March-Dec 2013 | 8.7 | 9.6 | -0.9** | 7.74 | 7.8 | -0.06 |
|  | (8.19, 9.21) | (9.02, 10.18) | (-1.41, -0.39) | (7.14, 8.33) | (7.45, 8.16) | (-0.80, 0.67) |
| Pre-tax period 2: March-Dec 2014 | 9.25 | 10.74 | -1.49** | 8.53 | 8.89 | -0.36 |
|  | (8.54, 9.95) | (10.17, 11.31) | (-2.57, -0.41) | (7.87, 9.19) | (8.47, 9.31) | (-1.43, 0.70) |
| Post-tax period:  March-Dec 2015 | 10.95 | 12.02 | -1.06 | 9.3 | 9.22 | 0.08 |
|  | (10.18, 11.73) | (11.21, 12.82) | (-2.42, 0.29) | (7.99, 10.62) | (8.54, 9.91) | (-1.80, 1.96) |
| *Absolute Difference (Posttax - Pretax2)* | *1.71*  *p=0.00* | *1.28**  *p=0.02* | *0.43*  *p=0.53* | *0.77*  *p=0.06* | *0.33*  *p=0.11* | *0.44*  *p=0.34* |
|  | *(0.85, 2.56)* | *(0.25, 2.31)* | *(-0.89, 1.75)* | *(-0.04, 1.59)* | *(-0.08, 0.75)* | *(-0.47, 1.35)* |
| **S9 Table continued** | | | | | | |
| **Plain waters** | **No taxed plain waters** | | | **Untaxed (N=337)** | | |
|  |  |  |  | **Berkeley** | **Non-Berkeley** | **Diff (B-NB)** |
| Pre-tax period 1: March-Dec 2013 |  |  |  | 1.91 | 1.86 | 0.05 |
|  |  |  |  | (1.78, 2.03) | (1.70, 2.01) | (-0.04, 0.14) |
| Pre-tax period 2: March-Dec 2014 |  |  |  | 2.12 | 1.98 | 0.14 |
|  |  |  |  | (1.84, 2.40) | (1.81, 2.16) | (-0.27, 0.55) |
| Post-tax period:  March-Dec 2015 |  |  |  | 2.1 | 2.07 | 0.03 |
|  |  |  |  | (1.85, 2.36) | (1.92, 2.21) | (-0.34, 0.41) |
| *Absolute Difference*  *(Posttax - Pretax2)* |  |  |  | *-0.02*  *p=0.76* | *0.08*  *p=0.07* | *-0.10*  *p=0.16* |
|  |  |  |  | *(-0.12, 0.09)* | *(-0.007, 0.17)* | *(-0.24, 0.04)* |
| **Plain milks** | **No taxed plain milks** | | | **Untaxed (N=337)** | | |
|  |  |  |  | **Berkeley** | **Non-Berkeley** | **Diff (B-NB)** |
| Pre-tax period 1: March-Dec 2013 |  |  |  | 4.31 | 4.3 | 0.01 |
|  |  |  |  | (4.16, 4.47) | (4.17, 4.44) | (-0.17, 0.19) |
| Pre-tax period 2: March-Dec 2014 |  |  |  | 4.83 | 4.73 | 0.1 |
|  |  |  |  | (4.64, 5.03) | (4.60, 4.86) | (-0.16, 0.37) |
| Post-tax period:  March-Dec 2015 |  |  |  | 4.85 | 4.66 | 0.19 |
|  |  |  |  | (4.29, 5.41) | (4.32, 5.01) | (-0.62, 0.99) |
| *Absolute Difference*  *(Posttax - Pretax2)* |  |  |  | *0.02*  *p=0.94* | *-0.06*  *p=0.75* | *0.08*  *p=0.79* |
|  |  |  |  | *(-0.41, 0.45)* | *(-0.45, 0.32)* | *(-0.50, 0.66)* |

Notes: **^‡^** Every barcode is weighted by their popularity (market share); N is the sample size of # of beverage categories sold from the nine stores per month in the POS data. Fixed Effects models account for the month-year (indicator variables), store located in Berkeley, interaction of Berkeley store and month-year, and an indicator variable of under-reported sales data from store in particular month. Prices account for inflation over time.

B=Berkeley; NB=non-Berkeley.

Jan-Feb of every year was excluded in the price comparisons due to the ambiguous period of Jan-Feb 2015 when the tax implementation was unclear since the original implementation date of Jan 1, 2015 was moved to March 1, 2015.

** denotes statistical significant difference between mean prices in the post-tax period (March-Dec 2016) from pre-tax period 2 (March-Dec 2014) at p<0.01, * denotes statistical significant difference between mean prices in the post-tax period (March-Dec 2016) from pre-tax period 2 (March-Dec 2014) at p<0.05.

Source: PHI Point-of-sales data from two chains of large supermarkets in the Bay Area.
