# Supplementary material for: Changes in prices, sales, consumer spending, and beverage consumption one year after a tax on sugar-sweetened beverages in Berkeley, California, US: A before-and-after study
Source: PLoS Med. 2017 Apr 18;14(4):e1002283. doi: 10.1371/journal.pmed.1002283 (PMC5395172; doi:10.1371/journal.pmed.1002283)
Supplement: S10 Table — CF, counterfactual; NB, non-Berkeley. (DOCX) [file pmed.1002283.s012.docx]

S10 Table Point-of-sales model adjusted monthly counterfactuals (CF^‡^) and observed sales of beverages in Berkeley vs non Berkeley (NB) stores, and mean absolute (oz/transaction) and relative (% of CF) differences

|  | **Berkeley** | | **Non-Berkeley (NB)** | | **NB Zone 1^§^** | | **NB Zone 2^§^** | | **NB Zone 3^§^** | |
| --- | --- | --- | --- | --- | --- | --- | --- | --- | --- | --- |
|  | **CF^‡^** | **Observed** | **CF^‡^** | **Observed** | **CF^‡^** | **Observed** | **CF^‡^** | **Observed** | **CF^‡^** | **Observed** |
| **Taxed Beverages** |  |  |  |  |  |  |  |  |  |  |
| Mar 2015 | 8.4 | 7.5 | 10.5 | 11.2 | 16.9 | 19.2 | 4.1 | 4.4 | 8.3 | 8.1 |
| April 2015 | 8.0 | 7.2 | 10.5 | 11.2 | 16.5 | 18.8 | 4.2 | 4.4 | 8.4 | 8.2 |
| May 2015 | 7.9 | 7.2 | 10.4 | 11.1 | 16.0 | 18.2 | 4.7 | 5.0 | 8.5 | 8.3 |
| June 2015 | 8.0 | 7.3 | 11.3 | 12.0 | 17.7 | 20.1 | 4.6 | 4.9 | 9.2 | 9.0 |
| July 2015 | 8.0 | 7.2 | 11.6 | 12.3 | 17.7 | 20.1 | 4.6 | 4.9 | 9.8 | 9.6 |
| Aug 2015 | 7.7 | 7.0 | 10.9 | 11.6 | 15.7 | 17.9 | 4.8 | 5.1 | 9.6 | 9.4 |
| Sept 2015 | 8.8 | 7.9 | 11.6 | 12.3 | 18.4 | 20.9 | 4.4 | 4.7 | 9.4 | 9.2 |
| Oct 2015 | 8.6 | 7.8 | 10.8 | 11.6 | 18.1 | 20.4 | 4.1 | 4.4 | 8.3 | 8.1 |
| Nov 2015 | 7.1 | 6.4 | 10.3 | 11.0 | 17.5 | 19.8 | 3.8 | 4.0 | 7.6 | 7.4 |
| Dec 2015 | 7.4 | 6.7 | 10.4 | 11.2 | 17.9 | 20.3 | 4.0 | 4.2 | 7.6 | 7.4 |
| Jan 2016 | 7.0 | 6.3 | 10.5 | 11.3 | 18.3 | 20.8 | 4.1 | 4.3 | 7.5 | 7.4 |
| Feb 2016 | 7.7 | 6.9 | 10.6 | 11.5 | 18.2 | 20.7 | 4.0 | 4.2 | 7.7 | 7.5 |
| *Mar 2015-Feb 2016, mean (95% CI)* | *7.9*  *(7.6, 8.2)* | *7.1*  *(6.9, 7.4)* | *10.8*  *(10.5, 11.0)* | *11.5*  *(11.2, 11.8)* | *17.4*  *(17.2, 17.6)* | *19.8*  *(19.6, 20.0)* | *4.3*  *(4.2, 4.4)* | *4.5*  *(4.5, 4.6)* | *8.5*  *(8.3, 8.7)* | *8.3*  *(8.1, 8.5)* |
| *Absolute diff, oz/trans* | *-0.76** (-0.8, -0.7)* | | *0.75** (0.7, 0.8)* | | *2.35** (2.3, 2.4)* | | *0.25** (0.25, 0.26)* | | *-0.17** (-0.17, -0.17)* | |
| *Relative diff, % of CF* | *-9.6%** (-9.9, -9.3)* | | *6.9%** (6.3, 7.2)* | | *13.5%** (13.1, 14.0)* | | *5.9%** (5.9, 5.9)* | | *-2.0%** (-2.2, -1.8)* | |
| **Untaxed Beverages** |  |  |  |  |  |  |  |  |  |  |
| Mar 2015 | 29.9 | 31.0 | 38.0 | 38.2 | 50.3 | 50.9 | 25.2 | 24.4 | 34.1 | 34.4 |
| April 2015 | 29.0 | 30.0 | 37.2 | 37.3 | 48.1 | 48.6 | 25.1 | 24.4 | 33.7 | 33.9 |
| May 2015 | 29.6 | 30.6 | 37.7 | 37.9 | 48.5 | 49.1 | 25.3 | 24.5 | 34.7 | 35.0 |
| June 2015 | 30.4 | 31.5 | 39.2 | 39.4 | 51.4 | 52.0 | 25.0 | 24.3 | 35.8 | 36.2 |
| July 2015 | 30.2 | 31.4 | 39.2 | 39.4 | 51.9 | 52.4 | 24.9 | 24.2 | 35.5 | 35.8 |
| Aug 2015 | 30.3 | 31.4 | 39.2 | 39.5 | 49.8 | 50.4 | 25.8 | 25.1 | 36.5 | 36.8 |
| Sept 2015 | 29.0 | 30.0 | 38.3 | 38.6 | 49.0 | 49.6 | 25.1 | 24.4 | 35.6 | 35.9 |
| Oct 2015 | 28.6 | 29.6 | 37.6 | 37.8 | 48.3 | 48.8 | 24.9 | 24.1 | 34.7 | 34.9 |
| Nov 2015 | 28.0 | 29.0 | 35.1 | 35.3 | 44.9 | 45.5 | 24.2 | 23.4 | 32.3 | 32.4 |
| Dec 2015 | 27.8 | 28.7 | 35.5 | 35.6 | 45.6 | 46.2 | 24.9 | 24.2 | 32.2 | 32.4 |
| Jan 2016 | 28.7 | 29.7 | 38.3 | 38.4 | 50.5 | 51.1 | 26.3 | 25.5 | 34.1 | 34.3 |
| Feb 2016 | 28.5 | 29.5 | 38.6 | 38.7 | 53.6 | 54.2 | 25.1 | 24.3 | 32.7 | 33.0 |
| *Mar 2015-Feb 2016, mean (95% CI)* | *29.2*  *(28.6, 29.8)* | *30.2*  *(29.5, 30.9)* | *37.8*  *(37.4, 38.3)* | *38.0*  *(37.5, 38.6)* | *49.33*  *(49.0, 49.7)* | *49.9*  *(49.5, 50.2)* | *25.1*  *(24.9, 25.4)* | *24.4*  *(24.2, 24.6)* | *34.3*  *(33.8, 34.9)* | *34.6*  *(33.9, 35.3)* |
| *Absolute diff, oz/trans* | *1.04** (0.90, 1.17)* | | *0.19** (0.10, 0.28)* | | *0.56** (0.48, 0.63)* | | *-0.74** (-0.75, -0.74)* | | *0.25** (0.09, 0.41)* | |
| *Relative diff, % of CF* | *3.5%** (3.1, 3.9)* | | *0.5%** (0.1, 0.9)* | | *1.1%** (0.8, 1.4)* | | *-3.0%** (-3.1, -2.9)* | | *0.7%** (0.5, 0.9)* | |
| **S10 Table continued** | | | | | | | | | | |
|  | **Berkeley** | | **Non-Berkeley (NB)** | | **NB Zone 1^§^** | | **NB Zone 2^§^** | | **NB Zone 3^§^** | |
|  | **CF^‡^** | **Observed** | **CF^‡^** | **Observed** | **CF^‡^** | **Observed** | **CF^‡^** | **Observed** | **CF^‡^** | **Observed** |
| **Taxed+Untaxed Beverages** |  |  |  |  |  |  |  |  |  |  |
| Mar 2015 | 38.3 | 38.7 | 48.5 | 49.2 | 67.4 | 69.4 | 29.2 | 28.8 | 42.3 | 42.5 |
| April 2015 | 36.9 | 37.1 | 47.7 | 48.4 | 65.0 | 66.9 | 29.2 | 28.9 | 41.9 | 42.1 |
| May 2015 | 37.6 | 37.8 | 48.2 | 48.8 | 65.0 | 66.9 | 29.9 | 29.5 | 43.0 | 43.2 |
| June 2015 | 38.5 | 38.7 | 50.5 | 51.3 | 69.6 | 71.7 | 29.5 | 29.2 | 44.9 | 45.0 |
| July 2015 | 38.4 | 38.6 | 50.8 | 51.5 | 69.9 | 71.9 | 29.4 | 29.1 | 45.2 | 45.4 |
| Aug 2015 | 38.1 | 38.4 | 50.2 | 50.9 | 65.9 | 68.0 | 30.5 | 30.1 | 45.9 | 46.1 |
| Sept 2015 | 37.7 | 37.9 | 49.8 | 50.6 | 67.6 | 69.7 | 29.4 | 29.1 | 44.8 | 45.1 |
| Oct 2015 | 37.2 | 37.4 | 48.3 | 49.0 | 66.4 | 68.3 | 28.9 | 28.6 | 42.8 | 43.0 |
| Nov 2015 | 35.2 | 35.4 | 45.3 | 46.0 | 62.5 | 64.4 | 27.8 | 27.5 | 39.7 | 39.9 |
| Dec 2015 | 35.2 | 35.4 | 45.9 | 46.5 | 63.7 | 65.7 | 28.7 | 28.4 | 39.7 | 39.8 |
| Jan 2016 | 35.7 | 35.9 | 48.9 | 49.6 | 69.3 | 71.3 | 30.2 | 29.9 | 41.5 | 41.7 |
| Feb 2016 | 36.2 | 36.5 | 49.2 | 50.0 | 72.2 | 74.2 | 28.9 | 28.6 | 40.2 | 40.4 |
| *Mar 2015-Feb 2016, mean (95% CI)* | *37.1*  *(36.2, 38.0)* | *37.4*  *(36.4, 38.4)* | *48.6*  *(47.9, 49.3)* | *49.3*  *(48.6, 50.1)* | *67.0*  *(66.6, 67.5)* | *69.0*  *(68.6, 69.4)* | *29.3*  *(29.1, 29.6)* | *29.0*  *(28.7, 29.2)* | *42.7*  *(41.9, 43.4)* | *42.9*  *(42.0, 43.8)* |
| *Absolute diff, oz/trans* | *0.28** (0.20, 0.35)* | | *0.71** (0.61, 0.81)* | | *1.98** (1.83, 2.13)* | | *-0.33** (-0.33, -0.33)* | | *0.20* (0.05, 0.35)* | |
| *Relative diff, % of CF* | *0.7%** (0.5, 0.9)* | | *1.5%** (1.2, 1.8)* | | *3.0%** (2.7, 3.3)* | | *-1.1%** (-1.2, -1.0)* | | *0.5%** (0.3, 0.8)* | |
| **# of store-day records in comparisons** | **1,090** | | **2,184** | | **732** | | **362** | | **1,090** | |

Notes: Models account for store ID, month, year, day of week, holiday and holiday-eve, number of transactions (linear and quadratic), an ambiguous period indicator, a post-tax indicator, and interacted the store ids with the ambiguous period, post-tax, and month variables, correcting the standard errors by clustering the analyses at the city level. Model N = 10,152

^‡^ CF= Estimated counterfactual volume based on pre-tax trends derived from predicting the volume if the post-tax indicator=0 during March 2015 through February 2016.

^§^ NB Zone 1 are non-Berkeley stores within the Bay Area closest to Berkeley; NB Zone 3 are non-Berkeley stores within the Bay Area furthest from Berkeley.

** denotes statistical significant difference between the counterfactual and observed volumes sold during the post-tax period at p<0.01, * denotes statistical significant difference between the counterfactual and observed volumes sold during the post-tax period at p<0.05.

Source: Point-of-sales (POS) data from chains of large supermarkets in the Bay Area.
