# Supplementary material for: Changes in prices, sales, consumer spending, and beverage consumption one year after a tax on sugar-sweetened beverages in Berkeley, California, US: A before-and-after study
Source: PLoS Med. 2017 Apr 18;14(4):e1002283. doi: 10.1371/journal.pmed.1002283 (PMC5395172; doi:10.1371/journal.pmed.1002283)
Supplement: S11 Table — CF, counterfactual; NB, non-Berkeley. (DOCX) [file pmed.1002283.s013.docx]

S11 Table Point-of-sales model adjusted counterfactuals (CF^‡^) and observed sales of untaxed beverage categories in Berkeley vs non-Berkeley (NB) stores, and mean absolute (oz/transaction) and relative (% of CF) differences

| **Untaxed beverage volume (oz per transaction) in March 2015-Feb 2016** | **Berkeley** | | | | **Non-Berkeley** | | | |
| --- | --- | --- | --- | --- | --- | --- | --- | --- |
|  | **CF ^‡^**  **mean**  **(95% CI)** | **Observed**  **mean**  **(95% CI)** | **Absolute Diff**  **mean**  **(95% CI)** | **Relative Diff, mean** | **CF ^‡^**  **mean**  **(95% CI)** | **Observed mean**  **(95% CI)** | **Absolute Diff mean**  **(95% CI)** | **Relative Diff, mean** |
| **Diet soft drinks & energy drinks** | 2.56  (2.53, 2.59) | 2.32  (2.30, 2.35) | -0.24 **  (-0.27, -0.21) | -9.22% | 2.53  (2.50, 2.56) | 2.60  (2.58, 2.62) | 0.08 **  (0.06, 0.09) | 2.96% |
| **Untaxed fruit, vegetable or tea drinks** | 5.13  (5.01, 5.25) | 5.35  (5.18, 5.53) | 0.22 **  (0.17, 0.28) | 4.37% | 6.09  (6.01, 6.16) | 6.49  (6.38, 6.60) | 0.40 **  (0.36, 0.44) | 6.52% |
| **Untaxed milk or substitute beverages** | 2.32  (2.27, 2.37) | 2.26  (2.22, 2.30) | -0.06 **  (-0.07, -0.05) | -2.53% | 2.52  (2.50, 2.55) | 2.55  (2.53, 2.57) | 0.03 **  (0.02, 0.03) | 1.09% |
| **Plain waters** | 7.72  (7.50, 7.95) | 8.93  (8.60, 9.28) | 1.21 **  (1.09, 1.34) | 15.62% | 12.82  (12.55, 13.13) | 12.26  (12.02, 12.53) | -0.56 **  (-0.63, -0.49) | -4.41% |
| **Plain milks** | 11.54  (11.36, 11.72) | 11.61  (11.42, 11.80) | 0.07 **  (0.07, 0.08) | 0.63% | 14.31  (14.13, 14.50) | 14.33  (14.14, 14.52) | 0.02 *  (0.001, 0.035) | 0.12% |

Notes: Models account for store ID, month, year, day of week, holiday and holiday-eve, number of transactions (linear and quadratic), an ambiguous period indicator, a post-tax indicator, and interacted the store ids with the ambiguous period, post-tax, and month variables, correcting the standard errors by clustering the analyses at the city level. Model N = 10,152

^‡^ CF= Estimated counterfactual volume based on pre-tax trends derived from predicting the volume if the post-tax indicator=0 during March 2015 through February 2016.

** denotes statistical significant difference between the counterfactual and observed volumes sold during the post-tax period at p<0.01, * denotes statistical significant difference between the counterfactual and observed volumes sold during the post-tax period at p<0.05.

Source: Point-of-sales (POS) data from chains of large supermarkets in the Bay Area.
