# Supplementary material for: Changes in prices, sales, consumer spending, and beverage consumption one year after a tax on sugar-sweetened beverages in Berkeley, California, US: A before-and-after study
Source: PLoS Med. 2017 Apr 18;14(4):e1002283. doi: 10.1371/journal.pmed.1002283 (PMC5395172; doi:10.1371/journal.pmed.1002283)
Supplement: S12 Table — (DOCX) [file pmed.1002283.s014.docx]

S12 Table Weighted frequency of reported consumption rates of taxed and untaxed beverages by subcategory pre- and post-tax, Berkeley California 2014-2015

|  | **Nov-Dec 2014** | | | **Nov-Dec 2015** | | |
| --- | --- | --- | --- | --- | --- | --- |
|  | **N Consumers** | **% Consumers** | **95% CI** | **N Consumers** | **% Consumers** | **95% CI** |
| **UNTAXED BEVERAGES** |  |  |  |  |  |  |
| Diet soda | 29 | 6.3 | 1.26-11.34 | 36 | 5.53 | 2.76-8.29 |
| Water | 564 | 87.81 | 79.70-95.92 | 559 | 92 | 88.59-95.42 |
| 100% Juice | 176 | 28.97 | 20.67-37.27 | 148 | 22.8 | 17.31-28.28 |
| Milk | 114 | 16.67 | 13.32-26.02 | 121 | 24.53 | 17.29-31.77 |
| Coffee/tea, unsweetened (before purchase) | 457 | 73.59 | 66.39-80.79 | 136 | 67.29 | 59.92-74.67 |
| Coffee/tea with sweetener | 78 | 7.61 | 5.16-10.06 | 127 | 17.76 | 12.78-22.75 |
| Flavored/sparkling water, unsweetened (before purchase) | 49 | 8.96 | 1.64-16.28 | 44 | 8.71 | 3.98-13.44 |
| Water with additions (e.g., fruit, juice) | 18 | 1.52 | 0.62-2.43 | 34 | 3.43 | 1.65-5.21 |
| Powdered drinks | 4 | 0.57 | 0.00-1.46 | 1 | 0.03 | 0.00-0.08 |
| Diet sports drinks | 0 | 0 | 0 | 2 | 0.09 | 0.00-0.22 |
| Diet energy drinks | 1 | 0.45 | 0.00-1.33 | 0 | 0 | 0 |
| Diet powdered drinks | 0 | 0 | 0 | 2 | 0.15 | 0.00-0.37 |
| Ready-to-drink diet iced teas | 1 | 0.07 | 0.00-0.22 | 4 | 1.21 | 0.00-2.85 |
| Diet Juice drinks | 0 | 0 | 0 | 2 | 0.16 | 0.00-0.37 |
| Other untaxed (e.g., yogurt smoothies, milkshakes, atole, horchata, non-alcoholic beer, eggnog, kefir) | 40 | 4.14 | 2.39-5.88 | 55 | 9.98 | 5.33-14.63 |
| **TAXED BEVERAGES** |  |  |  |  |  |  |
| Energy drinks | 2 | 0.61 | 0.00-1.46 | 1 | 0.35 | 0.00-1.04 |
| Juice drinks | 30 | 4.81 | 2.66-6.96 | 33 | 5.28 | 2.52-8.04 |
| Sports drinks | 4 | 1.71 | 0.00-4.07 | 5 | 1.23 | 0.00-2.49 |
| Regular soda | 73 | 14.81 | 9.25-20.37 | 53 | 11.32 | 5.96-16.68 |
| Ready-to-drink iced teas | 1 | 0.02 | 0.00-0.07 | 7 | 3.11 | 0.00-6.57 |
| Flavored/sparkling water, sweetened (before purchase) | 1 | 0.04 | 0.00-0.13 | 3 | 0.33 | 0.00-0.72 |
| Coffee/tea, sweetened (before purchase) | 14 | 1.65 | 0.26-2.74 | 19 | 3.97 | 1.34-6.60 |
| Other taxed (e.g., kombucha, aloe vera, hot chocolate) | 30 | 7.53 | 0.94-14.12 | 12 | 1.71 | 0.35-3.07 |

Notes: The present analysis utilizes self-reported 24h dietary recall data over two days
