# Supplementary material for: Changes in prices, sales, consumer spending, and beverage consumption one year after a tax on sugar-sweetened beverages in Berkeley, California, US: A before-and-after study
Source: PLoS Med. 2017 Apr 18;14(4):e1002283. doi: 10.1371/journal.pmed.1002283 (PMC5395172; doi:10.1371/journal.pmed.1002283)
Supplement: S13 Table — (DOCX) [file pmed.1002283.s015.docx]

S13 Table Descriptive statistics of Berkeley sample in 2014 and 2015 compared to adult sample (age 18y and older) in the National Health and Nutrition Examination Survey (NHANES), 2011-2012

|  | **Berkeley Dietary and Shopping Behavior Survey ^a^** | | |  | **NHANES ^e^** |
| --- | --- | --- | --- | --- | --- |
|  | 2014 (n=623) | 2015 (n=613) | *P* ^b^ |  | 2011-12 (n=4670) |
| Gender, No. (%) |  |  | 0.6 |  |  |
| Male | 287 (48%) | 255 (51%) |  |  | 2323 (49%) |
| Female | 345 (52%) | 358 (49%) |  |  | 2347 (51%) |
| Income, No. (%) |  |  | **0.01** |  |  |
| FPL ≤ 185 | 122 (30%) | 82 (16%) |  |  | 2330 (37%) |
| FPL >185 | 501 (70%) | 531 (84%) |  |  | 2340 (63%) |
| Education, No. (%) |  |  | 0.93 |  |  |
| High school or less | 70 (11%) | 51 (11%) |  |  | 2045(36%) |
| Some college | 100 (23%) | 90 (21%) |  |  | 1462 (33%) |
| College graduate | 453 (66%) | 472 (68%) |  |  | 1163 (31%) |
| Race/ethnicity, No. (%) |  |  |  |  |  |
| Non-Hispanic White | 428 (61%) | 446 (62%) | 0.86 |  | 1808 (67%) |
| Non-Hispanic Black | 65 (13%) | 56 (5%) | **0.01** |  | 1228 (11%) |
| Hispanic | 67 (12%) | 55 (15%) | 0.48 |  | 914 (14%) |
| Non-Hispanic Other | 63 (14%) | 56 (18%) | 0.43 |  | 720 (7%) |
| Age, mean (CI) | 43.9 (40.1, 47.7) | 42.4 (39.9, 45.0) | 0.54 |  | 46.0 (43.9, 48.1) |
| Taxed beverage^c^ consumption, No. (%) | 143 (29%) | 173 (32%) | 0.60 |  | 2847 (58%) |
| Untaxed beverage consumption, No. (%) | 621 (99.8%) | 609 (99.2%) | 0.05 |  | 4586 (98%) |
| Per capita intake of taxed beverages, kcal/day, mean (CI)^d^ | 48.2 (31.9, 64.5) | 42.4 (29.6, 55.2) | 0.58 |  | 131 (122, 142) |
| Per consumer intake of taxed beverages, kcal/day, mean (CI) | 220.1 (172.4, 267.9) | 201.1 (171.0, 231.2) | 0.50 |  | 271 (263, 281) |
| Per capita intake of untaxed beverages, kcal/day, mean (CI) | 110.4 (85.8, 135.0) | 142.8 (110.8, 174.8) | 0.12 |  | 130 (123, 137) |
| Per consumer intake of untaxed beverages, kcal/day, mean (CI) | 110.6 (85.9, 135.2) | 144.0 (111.8, 176.2) | 0.10 |  | 134 (127, 142) |

Notes: ^a^ Weighted to represent the Berkeley population; ^b^ *p*-value for proportions testing within group, 2014 vs. 2015; ^c^ Calculated based on two-day reported (observed intake). ^d^ Per capita intake is the mean average calories consumed by all survey participants (both consumer and non-consumer), whereas per consumer intake is the mean average calories consumed by only those participants who reported consuming that beverage;

^e^ NHANES is a nationally representative survey designed to examine the health and nutrition status of the United States population. The NHANES methodology has been described in detail elsewhere^19^.

The present analysis utilizes self-reported 24h dietary recall data over two days.
