# Supplementary material for: Changes in prices, sales, consumer spending, and beverage consumption one year after a tax on sugar-sweetened beverages in Berkeley, California, US: A before-and-after study
Source: PLoS Med. 2017 Apr 18;14(4):e1002283. doi: 10.1371/journal.pmed.1002283 (PMC5395172; doi:10.1371/journal.pmed.1002283)
Supplement: S1 Text — (DOCX) [file pmed.1002283.s016.docx]

S1 Text Details on the Store Price Surveys sample design, data collection procedures and price analysis

The Store Price Survey (SPS) was conducted in a targeted sample of large chain supermarkets (n=6); small chain supermarkets (n=2) and chain gas stations (n=2); pharmacies (n=2); and independent corner stores (n=13) and independent gas stations (n=1) located in Berkeley, CA (n=26). Three surveys were conducted in December 2014 (pre-tax), June 2015 (post-tax round 1), and March 2016 (post-tax round 2).

First, the sample of stores was selected using the top six stores where participants most frequently shopped, as reported in the Dietary and Shopping Behavior (DSB) telephone survey (See ***S5 Text***) question on where respondents reported most frequently purchasing beverages. Additional stores were added from random selection within their categories from a list of licensed businesses operating in the City of Berkeley to ensure that the sample included stores of different store types, and stores located in major commercial neighborhoods with businesses in Berkeley, including small grocers and grocers serving the Latino, African American and Middle Eastern communities. When stores refused to allow the data collectors to enter prices, a replacement store was assigned from the random selection of same store type and neighborhood.

Within each store, a standard panel of 70 beverage items was selected using information on top selling beverages in the Bay Area and nationally and representing the range of beverage categories (***S1 Table***). Additional beverages similar to beverages in the standard beverage panel were collected in a supplemental beverage panel; this was especially helpful in some stores that did not stock any beverages in the standard beverage panel. This panel included less well-known beverage brands, store brands and some other package types for large brands. All beverages in the SPS were categorized into: soda, energy drinks, juice drinks, sports drinks, ready-to-drink teas, powdered drinks, 100% juice, milk and water. For each beverage in the SPS, price was collected for regular and diet variations (when available), as well as for selected common container sizes when available in the stores. Because none of the stores sold all the beverages in our panel, not every beverage in our panel was collected for every store.

Prices were collected using a systematic, standardized process by trained data collectors from December 15 through December 28, 2014, from June 1 through June 17, 2015, and from March 2 through March 9, 2016. Trained data collectors entered pre-sales tax and pre-bottle fee price information into a database using a tablet computer and paper forms (December 2014) or only paper forms (June 2015 and March 2016). If price information was displayed on a shelf-tag or on the product, that price was recorded in the database. The same standardized data collection process was followed during all three time periods, but the tablet computers were not used during June 2015 and March 2016 because the data collection was more efficient and conducted more quickly using paper forms. The data collected on paper forms was double-entered by trained research project assistants into a database and results were compared. If any results were dissimilar, the values were compared against the value on the paper form and entered as appeared on the form.

In the pre-tax round conducted in December 2014, 744 prices were collected for beverages (560 for the standard beverage panel, and 184 for the supplemental beverage panel) (***S2 Table***). In the June 2015 round, 798 prices were collected (602 for beverages in the standard beverage panel, and 196 for supplemental beverages). In the March 2016 round, 633 prices were collected, of which 511 were of beverages in the standard beverage panel and 126 were supplemental items.

***S2 Table*** presents the number of various beverage products and types of stores included in the SPS in each round. Results present in ***Fig 2 of the main paper*** and ***S3 Table*** are limited only to items matched as to product and store in all three rounds, which comprised 55 products and 313 prices across stores. ***S4 Table*** presents the results on price changes when items from stores are matched for comparison over two rounds only. Absolute differences in price per ounce between taxed and untaxed beverages are also presented in ***S3 and S4 Tables***.
