# Supplementary material for: Changes in prices, sales, consumer spending, and beverage consumption one year after a tax on sugar-sweetened beverages in Berkeley, California, US: A before-and-after study
Source: PLoS Med. 2017 Apr 18;14(4):e1002283. doi: 10.1371/journal.pmed.1002283 (PMC5395172; doi:10.1371/journal.pmed.1002283)
Supplement: S2 Text — (DOCX) [file pmed.1002283.s017.docx]

S2 Text Details on the Point-of-sale data, and price, volume sold and store revenue analyses


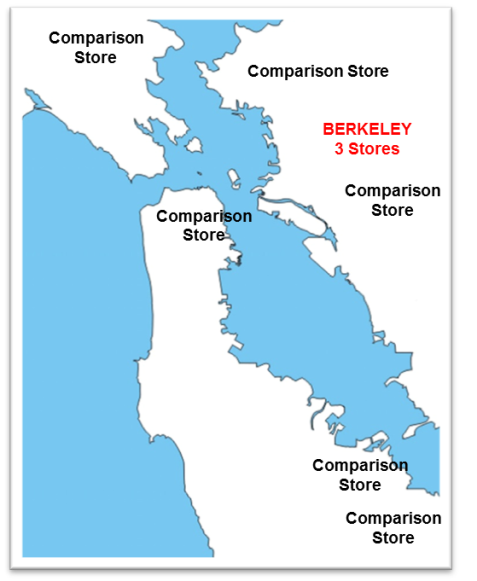
Point of sales (POS) data from retailers was requested using personal outreach to all large supermarkets in Berkeley (nine stores from six chains) as well as to pharmacy chains, small markets, ethnic markets, convenience stores, and gas stations, and accompanied by follow up with their respective managers, franchise owners or corporate headquarters. Fourteen chains with 30 stores and 5 independent markets with scanner systems were visited. Some did not have IT systems that could support the research – for example they might have a scanner but checked out customers without using it. The request was sensitivity because it involved information technology time and handing over confidential financial and business records with no benefit to the company. Outreach was continued in most cases until receiving a response, although in a few cases none was forthcoming. Compensation was offered but not accepted. Ultimately, two chains of large grocery stores generously agreed to participate and provided their electronic sales databases. No pharmacy, gas station or convenience store chains agreed to share their data. The two chains operated three of Berkeley’s nine large supermarkets, and shared data on six other supermarkets in the Bay Area, which served as comparison stores, as shown in the map. Demographic characteristics of store neighborhoods are described in ***S5 Table*.**

POS transaction data from these stores from January 1, 2013 through February 29, 2016 (24 months pre-tax; 2 month ambiguous; 12 months post-tax) was included in the analysis. In total, over this 38 month period across nine stores, the data consisted of 118.8 million barcode-scans from 15.5 million transactions or checkout episodes, with 16.2 million of these barcode-scans including beverages (16,769 unique barcodes), of which 10.8 million barcode-scans (5,631 unique barcodes) are included here.

Every beverage was placed in one of five mutually exclusive categories: 1) Soft drinks and energy drinks; 2) Fruit, vegetable and tea drinks; 3) flavored milk or substitute beverages 4) plain waters; and 5) plain milks. The first three categories contain both taxed and untaxed items, while the latter two categories are untaxed. Classification of tax status followed the Berkeley law^1^ and best as possible based on available data from product websites and ingredient data from Mintel,^2^ a database that contains information about the nutrition labels, ingredient lists and nutrient or health claims on packaged products starting in 1996 to date.

***S6 Table*** provides details on the various types of barcode-scans captured and what portion are included in this study, and ***S7 Table*** provides a description of products included in this study. The exclusion of nearly 5.4 million beverage-related barcode-scans from 11,138 beverage-type items were due to beverages that were not included in the study. Excluded beverage products were: Ground/ whole bean coffee; tea bags/loose tea; Liquid and powder concentrates/mixes; Alcohol (beer, wine, liquor, mixed alcoholic beverages); Meal replacements; Medical/dietary supplements; Infant formula; Any product added to another beverage (e.g., syrups, creamers); and Ready-to-drink fountain or hot drinks with no objective size information (e.g., descriptors are “small/medium/large”). Powders and concentrates were not covered by the law in groceries, although initially the interpretation was unclear.

To put the POS data in context, we used a representative sample of food purchases for the Bay Area from the 2014 Nielsen Homescan data^3,4^ in a separate descriptive analysis. We found that about 50% of the volume of beverages are from chain supermarkets (defined as ≥10 locations nationwide), 26% of beverages are from club or warehouse stores (e.g., Sam’s club, Costco, BJ’s), 7% are from drug and convenience stores (e.g., 7-eleven, CVS), 11% are from mass merchandisers (e.g., Target, Walmart), and about 2% are from independent stores (<10 locations nationally) [authors own calculations based on 2014 Nielsen Homescan data]. The two chains for which we have POS data account for three of the nine chain supermarkets (from six chains) located in Berkeley. The other six stores for which we have POS data are used as comparisons based on geographical proximity to Berkeley. Berkeley does not have any club or warehouse stores though they exist in neighboring communities. The geographical proximity analysis was added to the analysis plans only after we had confirmation of voluntarily participating chains and locations.
