# Supplementary material for: Changes in prices, sales, consumer spending, and beverage consumption one year after a tax on sugar-sweetened beverages in Berkeley, California, US: A before-and-after study
Source: PLoS Med. 2017 Apr 18;14(4):e1002283. doi: 10.1371/journal.pmed.1002283 (PMC5395172; doi:10.1371/journal.pmed.1002283)
Supplement: S3 Text — (DOCX) [file pmed.1002283.s018.docx]

S3 Text Point-of-sales Price Fixed Effects models and predictions

With the POS data from two supermarket chains, we were interested in both the degree to which the inflation-adjusted prices of taxed beverages changed among these stores, as well as when the price changes occurred. The POS data includes repeated measures of beverages sold (at barcode level) from both Berkeley and non-Berkeley stores, during both pre-tax, “ambiguous”, and post-tax periods.

**Sales-unweighted price changes**

We first looked at the price changes for each barcoded beverage item included in the study. The analysis does not take into account the fact that beverage items vary in popularity (have different market share), so we call these sales-unweighted price changes.

We used a Fixed Effects approach with inflation-adjusted price (cents/oz) of taxed beverages at the barcode-month-store level as the outcome, controlling for the month-year (relative to January 2013), interacting Berkeley store with the month-year dummies, and adjusting for potential under-reporting due to data missing completely at random on random days (due to technical issues in data storage identified after preliminary data collection) from some stores in certain months. The model specification was:

$${Price}_{ismy}=\beta_{M}M_{my}+\beta_{B}{Berkeley}_{s}+\beta_{MB}\left( {M_{my}*Berkeley}_{s} \right)+ {\vartheta UnderReport}_{smy}+{\mu_{is}+ \varepsilon_{ismy}},$$

where *Price* is the price (cents/oz) of product *i* sold in store *s* in month-year *my*. *M* denote the vector of month-year indicators in month *m* within year *y*, *Berkeley* denote store *s* being located in Berkeley, *UnderReport* denote an indicator for store-month specific underreported (due to random missingness) data. *µ* denotes product-store level fixed effects, and *ε* denotes the error term.

The coefficients of the interacted variables, *β_MB_* tell us the difference in sales-unweighted price per ounce of taxed beverages in Berkeley compared to non-Berkeley stores in each month-year relative to January 2013. For interpretation, we derive sales-unweighted adjusted price (cents/oz) of taxed and untaxed beverages sold from Berkeley vs non-Berkeley stores and present these. Since there was some ambiguity around the tax implementation, we kept data from January 2015 and February 2015 in our analyses, but excluded them in the comparisons of our results. Thus, we compared the sales unweighted adjusted price (cents/oz) in the similar months preceding the tax to the after the tax was unambiguously in place (i.e., March 2014-Dec 2014 vs March 2015-Dec 2015) and conducted statistical tests. We did this overall and by beverage categories as well as by package sizes. All analyses were conducted in Stata 13.^5^

Results are shown in ***S8 Table***, and ***Fig 3 of the main paper*** illustrates how the sales-unweighted prices changed over the entire period of the available data. From ***S8 Table*** we see that within the Berkeley stores, taxed beverages had price change of +0.83 cents/oz (*p*=0.00), while untaxed beverage prices rose 0.32 cents/oz (*p*=0.00), for a net difference of +0.51 cents/oz (*p*= 0.00, not reported in table due to space). This is in comparison to the Store Price Survey results from large supermarkets of +1.07 cents/oz (taxed beverages) and +0.51 cents/oz (untaxed beverages) for a net difference of +0.56 cents/oz (*p*=0.22) (See ***Fig 2 of the main paper/S3 Table***).

The frequency of the POS data allows us to document the monthly changes in prices, and we see in ***Fig 3 of the main paper*** that there were price increases after January 2015, but it was not until around April 2015 that prices stabilized. With data from Non-Berkeley stores within the Bay Area, we are also able to compare how prices changed in comparison locations. We found that there were some price increases for both taxed and untaxed beverages in Non-Berkeley stores as well, but the net price change among taxed beverages was still higher in Berkeley stores by 0.67 cents/oz (p=0.00) and no different between Berkeley and Non-Berkeley stores for untaxed beverages.

Lastly, with the POS data, we were able to look at price changes across the beverage categories, which show that among taxed beverages sold in these two chains of large supermarkets, the largest price changes were for soda and energy drinks, followed by fruit, vegetable or tea drinks, and flavored milk or substitute beverages had the lowest price increases. Meanwhile price changes among untaxed beverages were negligible and non-significant. Results by package sizes are not presented by available upon request.

**Sales-weighted price changes**

Next, we took into account sales of each beverage item within each beverage category as shown in ***S9 Table*** to measure sales-weighted prices for each store during each month. Again we used a Fixed Effects approach and the same model specification as for the sales-unweighted models, except that *i* now denotes beverage category (not product). The interpretation of the results are similar but are in terms of sales-weighted prices.

As expected, we found that in general sales-weighted prices are lower than sale-unweighted prices since consumers buy more of the lower priced items. In the POS sales-weighted, POS sales-unweighted analyses, as well as in the Store Price Survey, we saw that taxed beverages are on average more expensive than untaxed beverages. ***S9 Table*** also shows that the net sales-weighted price change among taxed beverages was higher in Berkeley stores by 0.65 cents/oz (*p*=0.003) and no different between Berkeley and Non-Berkeley stores for untaxed beverages, which is consistent with the sale-unweighted findings. Similarly, in the sales-weighted analyses, we found that among taxed beverages sold in these two chains of large supermarkets, the largest price changes were for soda and energy drinks, but there were no statistical changes in the prices of fruit, vegetable or tea drinks, and flavored milk or substitute beverages. Meanwhile price changes among untaxed beverages were generally negligible and non-significant, with the exception of untaxed sodas and energy drinks.
