# Supplementary material for: Changes in prices, sales, consumer spending, and beverage consumption one year after a tax on sugar-sweetened beverages in Berkeley, California, US: A before-and-after study
Source: PLoS Med. 2017 Apr 18;14(4):e1002283. doi: 10.1371/journal.pmed.1002283 (PMC5395172; doi:10.1371/journal.pmed.1002283)
Supplement: S4 Text — (DOCX) [file pmed.1002283.s019.docx]

S4 Text Point-of-sales volume sold difference-in-difference models and predicted outcomes

To determine changes in the volume of taxed and untaxed beverages sold, we used the Point-of-sales scanner data from two large supermarkets chains at the store-day level across the nine stores (three in Berkeley; six outside Berkeley) from 1 January 2013 through 29 February 2016. In reviewing the data from these chains, we noticed that some days of data were missing and followed up with the retailers about this. We were told that there may have been a few data or software glitches in their system from time to time. Therefore, we assume that these are missing at random. In total, over this 38 month period across nine stores, there were a total of 10,152 store-day observations. ***S1 Fig*** shows the mean volume of taxed and untaxed beverages sold per transaction in Berkeley vs non-Berkeley stores.

Map courtesy of http://d-maps.com/conditions.php?lang=en

Even though the Berkeley SSB tax was limited to the city of Berkeley, using other non-Berkeley stores as comparisons has certain limitations because: a) it is possible that shoppers shifted to shopping outside Berkeley in response to the tax, and; b) the Berkeley vs non-Berkeley stores may serve different populations and may face different types of competition.


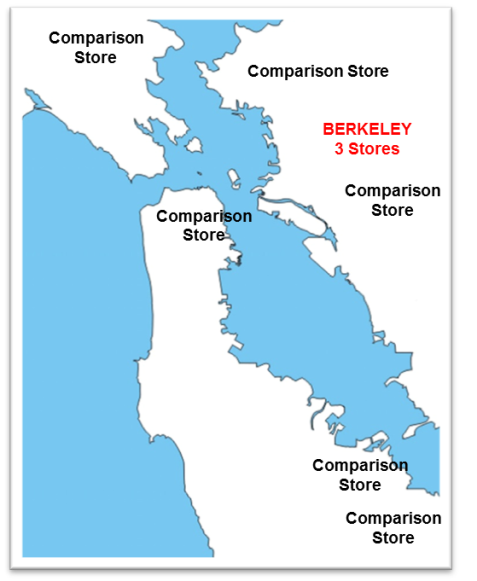


Zone 1

Zone 2

Zone 3

To handle the first issue, beyond comparing Berkeley to non-Berkeley stores, we also looked at changes in the beverage bought from non-Berkeley stores based on distance to Berkeley. We hypothesize that stores located in cities adjacent to Berkeley were more likely to experience ‘leakage’ (or spill-over effects) of the tax in Berkeley. We identified three non-Berkeley ‘zones’ with Zone 1 being stores located in cities adjacent to Berkeley (two stores in two adjacent cities), Zone 2 being stores located in San Francisco (one store), and Zone 3 being stores located in the three cities ≥20 miles from Berkeley (three stores). These three non-Berkeley store zones are illustrated on the right.

To handle the second issue, we applied a pre-post quasi-experimental approach that includes indicator variables for each store and in essence perform a fixed-effects model.^6,7^ We wanted to determine whether there were significant changes in beverage volume sold and total store revenue in Berkeley and non-Berkeley stores during the post-tax period compared to the pre-tax period while controlling for potential unmeasured store-specific factors (e.g., the clientele they serve, their store size, etc.). We constructed a counterfactual for what the sold volumes and store revenues in the post-tax period would have looked like in the absence of the tax and compared the observed post-tax sales volumes and store revenues to their counterfactual, holding all other factors constant.

**Beverage volume sold to shoppers**

The distribution of the both volume of beverages sold and their residuals were skewed (typically positively skewed) and not normally distributed (based on untransformed models), so we used the logarithm (log) of beverage sales as outcomes. The continuous explanatory variables and their residuals were more normally distributed and did not require any transformations. The model specification was:

$${log(BEV}_{sdmy})= \beta_{S}{StoreID}_{s}+\beta_{Y}{Year}_{y}+\beta_{M}{Month}_{m}+\beta_{T}{Posttax}_{my}+\beta_{SY}{StoreID}_{s}*{Year}_{y}+\beta_{SM}{StoreID}_{s}*{Month}_{m}+\beta_{ST}{StoreID}_{s}{*Posttax}_{my}+\alpha{Dayofweek}_{d}+\delta{Holiday}_{dmy}+ {\vartheta HolidayEve}_{dmy}+\gamma{Transaction}_{sdmy}+\varphi{Transaction}_{sdmy}^{2}{+ \varepsilon_{sdmy}}$$

The outcomes were the log of the volume of beverage *BEV* sold by store *s* on day *d* nested within month *m* and year *y*. Since we wanted results across Berkeley and non-Berkeley stores by zone, we ran parsimonious Ordinary Least Squares models for each outcome that controlled for time invariant factors of each store (e.g., size, location) by including indicator variables (*StoreID*). We also included dummy measures for month and year to account for potential seasonality and annual secular trends. The *Posttax* indicator variable indicates when observations are from 1 March 2015 and later. The *StoreID*Month* and *StoreID*Year* measures account for time-varying factors for each store such as the addition or departure of other stores, or changes in the demography of the clientele over time. Since shopping behaviors vary over the week (weekends are peak grocery shopping days) as well as around holidays, we controlled for these factors in the model. Additionally, we accounted for the number of transactions each store had on any given day, allow for both a linear and quadratic relationship to the outcome. We considered using volume per transaction as an outcome, but the distribution was highly bimodal, therefore we determined it was best to correct for the number of transactions as explanatory variables instead. The *StoreID*Posttax* interactions were included so that we could estimate the average effect of the tax (relative to a referent store) holding all else constant, and *ε* denotes the time-varying error. We corrected the standard errors by clustering the analyses at the city level (stores were located in seven different cities). We conducted all analyses with Stata 13.^5^

To allow for interpretability in these coefficients, we back-transformed the logged outcomes by calculating and applying Duan smearing factors^8^ and then divided this by the number of transactions. Specifically, Duan smearing ensures that in the presence of nonzero variances in the volume sold, the back-transformed predicted outcome is not downward biased.^8^ We do this for Berkeley and non-Berkeley stores, as well as for the non-Berkeley stores in each of the three zones. Additionally, we predicted the volume of taxed and untaxed beverages sold if *Posttax*=0 during March 2015 through February 2016 (i.e., a counterfactual for if the tax has not been implemented). This also allowed us to compare in absolute and relative terms the estimated post-tax volume sold in March 2015 through February 2016 to the estimated counterfactual post-tax volume sold.

***Fig 4 in the main paper*** illustrates the key findings, and ***S10 Table*** and ***S11 Table*** provide the detailed results.

**Store revenue or consumer spending ($ per Transaction)**

We ran a similar model for the store revenue per transaction outcome, but excluded the number of transactions as controls and did not need to log-transform the outcome (inflation-adjusted dollars per transaction) as that was normally distributed. The model specification was:

$${REVPerTrans}_{sdmy}= \beta_{S}{StoreID}_{s}+\beta_{Y}{Year}_{y}+\beta_{M}{Month}_{m}+\beta_{T}{Posttax}_{my}+\beta_{SY}{StoreID}_{s}*{Year}_{y}+\beta_{SM}{StoreID}_{s}*{Month}_{m}+\beta_{ST}{StoreID}_{s}{*Posttax}_{my}+\alpha{Dayofweek}_{d}+\delta{Holiday}_{dmy}+ {\vartheta HolidayEve}_{dmy}{+ \varepsilon_{sdmy}}$$

Again, we predicted the outcomes for Berkeley and non-Berkeley stores, as well as for non-Berkeley stores and compared in absolute and relative terms the estimated post-tax store revenue in March 2015 through February 2016 to the estimated counterfactual post-tax store revenue. ***Fig 5 in the main paper*** illustrates the key findings.
