# Supplementary material for: Changes in prices, sales, consumer spending, and beverage consumption one year after a tax on sugar-sweetened beverages in Berkeley, California, US: A before-and-after study
Source: PLoS Med. 2017 Apr 18;14(4):e1002283. doi: 10.1371/journal.pmed.1002283 (PMC5395172; doi:10.1371/journal.pmed.1002283)
Supplement: S5 Text — (DOCX) [file pmed.1002283.s020.docx]

S5 Text Details on the Dietary and Shopping Behavior Survey

The Dietary and Shopping Behavior Telephone Surveys were conducted in Nov-Dec 2014 (pre-tax) and Nov-Dec 2015 (post-tax). The sample was identified using dual frame (landline/cellular) random-digit-dialing with oversampling of lower income census blocks (>50% of households with annual gross household income <$100,000) in the city of Berkeley. Cellular phone sample was pulled from the 2 rate centers with the highest rates of users in Berkeley. Only those who were residents of Berkeley at the time of the survey were interviewed. Trained interviewers conducted interviews using standardized questionnaires and computer-assisted telephone interviewing software to collect information on 24-hour beverage recall, shopping behaviors, and demographics^9^. To adjust for typical daily intake, a second 24-hour beverage recall interview was collected three to seven days after the first interview with respondents who consented.

We had proposed a sample size of 1,155, with stratified samples of white (n=385), Latino/ Hispanic (n=385), and African American (n=385) residents to allow sufficient power comparisons by race/ethnicity. We expected that the tax would lower soda consumption by at least 10%, as suggested by a group of researchers at the University of California, San Francisco^10^, and that our study would have 80% power to detect a reduction of at least 10% in soda among Berkeley residents. Power analysis done using proc power from SAS statistical software using 0.7 as expected value for sigma. Given the limited time-frame (between the vote passing in Nov 2014 and the original implementation date of Jan 1, 2015), combined with major holidays (Thanksgivings, Christmas and New Year’s), we were only able to obtain a baseline sample of 649 prior to the original implementation date. We were also unable to obtain sufficient Latino and Hispanic respondents or low-income respondents for the original desired stratified analysis despite using full call center capacity and all available resources. By the time it was announced that the tax implementation was going to be delayed until March 1, 2015 (announced on Jan 26, 2015) it was too late to try to reassemble the survey team to add to the baseline sample. Subsequent collaborations with operators providing telephone survey research support in California, especially in the Bay area, have confirmed the challenges they are now facing in obtaining responses, particularly with minority respondents, and post-elections, and the need for a longer time to roll out samples.

At the baseline DSB (Nov-Dec 2014), 649 Berkeley residents ages 18 and older participated, of which 253 also completed a second 24-hr beverage recall. At the follow-up DSB (Nov-Dec 2015), 654 Berkeley residents participated, of which, 456 also completed a second 24-hr beverage recall; 346 (53.3%) of the respondents at follow-up completed the DSB baseline survey. For eligible respondents who were reached in 2014 (baseline), our cooperation rate was 17.4% (649/ 3721 eligible adults). For new eligible respondents who were reached in 2015 our cooperation rate was 20.9%. For panel members followed up in 2015, 346 of 412 eligible 2014 participants reached responded; AAPOR response rates are 73.7% and cooperation rates are 90.8% for the panel. Sampling weights were calculated using iterative proportional fitting (raking)^11^ to adjust the data to demographic proportions (age, gender, race/ethnicity) for Berkeley, California obtained from the United States Census Bureau 2010.^11,12^ Reported beverages consumed were converted into caloric intakes using data from websites, nutrition facts panel data from Mintel^2^, and USDA databases.^13,14^ Powdered beverages and concentrates were adjusted for volume following preparation instructions.

The questionnaire collected information on 24-hour beverage intake, shopping habits, soda intake habits, demographics (age, gender, race/ethnicity, and household size), and socioeconomic status (poverty level and education). Questions were adapted from the Behavioral Risk Factor Surveillance System and the California Dietary Practices Survey, when possible, because these questions have been cognitively tested and are recognized as valid and reliable. Participants reported the exact type of beverage consumed, including product brand information where available. Calorie content was assigned to each drink based on product websites where available or according to the USDA National Nutrient Database for Standard Reference, as were beverage subcategories, and taxation status (yes/no according to beverage category and whether the beverage contained added sugar, according to the details of the law). ***S11 Table*** shows taxed and untaxed beverage subcategories and the proportion of respondents reporting consuming each of these at baseline and at follow-up. It also illustrates the high proportion of non-consumption (self-report) of a number of these beverage subcategories, and hence our inability to adjust for usual intake (as described in ***S6 Text***) at the subcategory level.

In the analyses, participants were included if they had non-missing values on self-reported race/ethnicity, age, education, and income and monthly intake of SSBs. The final analytic sample included 623 at baseline and 613 at follow-up.

***S12 Table*** presents the descriptive statistics on the sample in 2014 and 2015 compared to adult sample (age 18y and older) in the National Health and Nutrition Examination Survey (NHANES), 2011-2012 without any adjustments for socio-demographic measures or adjusting for usual intake (as described in ***S6 Text***).
