# Supplementary material for: Changes in prices, sales, consumer spending, and beverage consumption one year after a tax on sugar-sweetened beverages in Berkeley, California, US: A before-and-after study
Source: PLoS Med. 2017 Apr 18;14(4):e1002283. doi: 10.1371/journal.pmed.1002283 (PMC5395172; doi:10.1371/journal.pmed.1002283)
Supplement: S6 Text — (DOCX) [file pmed.1002283.s021.docx]

S6 Text Usual intake modeling approach

This study employed the National Cancer Institute (NCI) method to estimate the distribution of usual intake (kcal and g) of taxed and untaxed beverages in each year,^15-18^ which takes advantage of multiple days of 24-h recalls to reduce within-person variability and estimate the distribution of longer-term intake of a food group in a population. In addition, for taxed beverages, a two-part model is used to account for the large proportion of non-consumers of taxed beverages. Because there has not yet been a validated method for longitudinal analysis using the NCI method, data was treated as repeated cross-sectional in order to retain a larger sample size and harness the advantage of the usual intake method to reduce within-person variability.

**Part I. Probability of consuming a beverage**

*Logit(24H Probability, Beverage_iy_) = α_I_ + β_I_Race/ethnicity + γ_I_Income + δ_I_Education + θ_I_Age + λ_I_Sex + π_I_DayofWeek + η_I_Year +Person-Specific Effect_I_*

Where subscript I indicates that these parameters are associated with Part I. The person-specific effect allows for each individual’s consumption probability to differ from the population level (i.e., the individual’s tendency to consume that beverage).

**Part II. Amount of beverage consumed**

*Transformed 24H Amount, Beverages= α_II_ + β_II_Race/ethnicity + γ_II_Income + δ_II_Education + θ_II_Age + λ_II_Sex + π_II_DayofWeek + η_II_Year +Person-Specific Effect_II_ + Within-person Variability_II_*

Where subscript II indicates that these parameters are from Part II, which models the amount of food consumed on the consumption day. The person-specific effect again allows for individual consumption amount to differ from the population level, while the within-person variability parameter allows for daily variation in an individual intake as well as other random error.

**Estimating distribution of Usual Intake**

Part I and Part II are linked by fitting both models simultaneously, with the person-specific effects modeled as correlated random variables, and overlapping covariates (race, income, education, sex, and day of recall) introducing additional correlation. This simultaneous estimation takes into consideration that an individual who is more likely to consume a beverage may also be more likely to consume a greater amount of that beverage on days when the beverage is consumed.^15^ For untaxed beverages, which are ubiquitously consumed, only the amount of beverage consumed is estimated, since the probability of consumption is nearly 100%. In both cases, the model is fit using maximum likelihood estimation, with a Box-Cox transformation to transform the 24-h amount to normality.

The NCI DISTRIB macro is then used to estimate the distribution of usual intake from the estimated parameters of the model.^19^ This macro simulates a population that has the same characteristics (as described by the values of the covariates) as the sample used to fit the model and uses a Monte Carlo method to empirically estimate the distribution (i.e., mean and percentiles) of usual intake. Bootstrapping with replacement was implemented 200 times to derive estimates of the standard errors for the beta coefficients in the model as well as the means and percentiles, from which p-values were calculated to test for significance among covariates in the model.

***Table 1 in the main paper*** provides the key findings after adjusting for both usual intake and socio-demographic measures in terms of kcals/capita/day as well as grams/capita/day. Standard errors were estimated via bootstrapping by drawing 200 random samples with replacement based on whether the individual responded to the 2014 survey only, 2015.
