# Supplementary material for: Changes in prices, sales, consumer spending, and beverage consumption one year after a tax on sugar-sweetened beverages in Berkeley, California, US: A before-and-after study
Source: PLoS Med. 2017 Apr 18;14(4):e1002283. doi: 10.1371/journal.pmed.1002283 (PMC5395172; doi:10.1371/journal.pmed.1002283)
Supplement: S7 Text — (DOCX) [file pmed.1002283.s022.docx]

S7 Text References for Online Supplements

**1.** Berkeley City Council. Imposing a General Tax on the Distribution of Sugar-Sweetened Beverage Products. In: Council BC, ed: Berkeley City Council; 2014.

**2.** Mintel Global New Product Database. <http://www.mintel.com/gnpd>.

**3.** Stern D, Poti JM, Ng SW, Robinson WR, Gordon-Larsen P, Popkin BM. Where people shop is not associated with the nutrient quality of packaged foods for any racial-ethnic group in the United States. *Am J Clin Nutr.* February 24, 2016 2016;103(4):1125-1134.

**4.** Stern D, Robinson WR, Ng SW, Gordon-Larsen P, Popkin BM. US household food shopping patterns: Dynamic shifts since 2000 and socioeconomic predictors. *Health Aff.* November 1, 2015 2015;34(11):1840-1848.

**5.** *Stata Statistical Software: Release 13* [computer program]. College Station, TX: StataCorp LP.; 2014.

**6.** Athey S, Imbens GW. Identification and inference in nonlinear difference-in-differences models. *Econometrica.* 2006;74(2):431-497.

**7.** Donald SG, Lang K. Inference with difference-in-differences and other panel data. *Rev Econ Stat.* 2007/05/01 2007;89(2):221-233.

**8.** Duan N. Smearing estimate: A nonparametric retransformation method. *J Am Stat Assoc.* 1983;78(383):605-610.

**9.** California Department of Public Health. Background on the California Dietary Practices Survey (CDPS). In: California Department of Public Health, ed: California Department of Public Health, ; 2013.

**10.** Wang YC, Coxson P, Shen Y, Goldman L, Bibbins-Domingo K. A penny-per-ounce tax on sugar-sweetened beverages would cut health and cost burdens of diabetes. *Health Aff.* 2012;31(1):199-207.

**11.** Izrael D, Hoaglin D, Battaglia M. A SAS macro for balancing a weighted sample, Paper #258-25. In: SAS, ed. *SAS Users Group International Conference, Statistics and Data Analysis*: SAS; 2000.

**12.** Quick Facts for Berkeley, California. 2010. <http://www.census.gov/quickfacts>.

**13.** USDA, ARS. USDA National Nutrient Database for Standard Reference. April 1, 2014 2014;Nutrient Data Laboratory Home Page, <http://www.ars.usda.gov/ba/bhnrc/ndl>.

**14.** Ahuja JKA, Montville JB, Omolewa-Tomobi G, et al. USDA Food and Nutrient Database for Dietary Studies, 5.0. U.S. . In: Department of Agriculture ARS, Food Surveys Research Group, ed. Beltsville, MD 2012.

**15.** Tooze JA, Midthune D, Dodd KW, et al. A new statistical method for estimating the usual intake of episodically consumed foods with application to their distribution. *J Am Diet Assoc.* 2006;106(10):1575-1587.

**16.** Tooze JA, Kipnis V, Buckman DW, et al. A mixed-effects model approach for estimating the distribution of usual intake of nutrients: the NCI method. *Stat Med.* 2010;29(27):2857-2868.

**17.** Belotti F, Deb P, Manning WG, Norton EC. twopm: Two-part models. *Stata J.* 2015;15(1):3-20.

**18.** Haines PS, Popkin BM, Guilkey DK. Modeling food consumption decisions as a two-step process. *Am J Agr Econ.* August 1, 1988 1988;70(3):543-552.

**19.** National Cancer Institute Division of Cancer Control and Population Sciences. Usual Dietary Intakes: SAS Macros for Analysis of a Single Dietary Component, SAS Macros Version 2.1. <http://epi.grants.cancer.gov/diet/usualintakes/macros_single.html>. Accessed July 1, 2015.
